# Endothelial progenitor cell-derived exosomes promote anti-inflammatory macrophages via SOCS3/JAK2/STAT3 axis and improve the outcome of spinal cord injury

**DOI:** 10.1186/s12974-023-02833-7

**Published:** 2023-06-30

**Authors:** Feifei Yuan, Wei Peng, Yuying Yang, Jiaqi Xu, Yudong Liu, Yong Xie, Tingmo Huang, Chaoran Shi, Yinghe Ding, Chengjun Li, Tian Qin, Shanshan Xie, Fengzhang Zhu, Hongbin Lu, Jianjun Huang, Jianzhong Hu

**Affiliations:** 1 Department of Spine Surgery and Orthopaedics, Xiangya Hospital, Central South University, Changsha, 410008 China; 2 Department of Sports Medicine, Xiangya Hospital, Central South University, Changsha, 410008 China; 3 Key Laboratory of Organ Injury, Aging and Regenerative Medicine of Hunan Province, Changsha, 410008 China; 4 Hunan Engineering Research Center of Sports and Health, Changsha, 410008 China; 5 National Clinical Research Center for Geriatric Disorders, Xiangya Hospital, Central South University, Changsha, 410008 China; 6 Department of Spine Surgery, Ningde City Hospital, Fujian Medical University, Ningde, China; 7 Department of Spine Surgery, Wuxi Ninth People’s Hospital, Wuxi, Jiangsu China

**Keywords:** Exosomes, Spinal cord injury, Endothelial progenitor cells, Pro-inflammatory macrophages, Anti-inflammatory macrophages, miR-222-3p, SOCS3

## Abstract

**Background:**

Macrophage in the spinal cord injury (SCI) area imparts a chronic pro-inflammation effect that challenges the recovery of SCI. Previously, endothelial progenitor cell-produced exosomes (EPC-EXOs) have been noticed to facilitate revascularization and inflammation control after SCI. However, their effects on macrophage polarization remained unclear. This study aimed to investigate the EPC-EXOs' role in macrophage polarization and reveal its underlying mechanism.

**Methods:**

We extracted the macrophages and EPC from the bone marrow suspension of C57BL/L mice by centrifugation. After cell identification, the EPC-EXOs were collected by ultra-high-speed centrifugation and exosome extraction kits and identified by transmission electron microscopy and nanoparticle tracking analysis. Then, macrophages were cultured with EPC-EXOs in different concentrations. We labeled the exosome to confirm its internalization by macrophage and detected the macrophage polarization marker level both in vitro and in vivo. We further estimated EPC-EXOs' protective effects on SCI by mice spinal cord tissue H&E staining and motor behavior evaluation. Finally, we performed RT-qPCR to identify the upregulated miRNA in EPC-EXOs and manipulate its expression to estimate its role in macrophage polarization, SOCS3/JAK2/STAT3 pathway activation, and motor behavior improvement.

**Results:**

We found that EPC-EXOs decreased the macrophages’ pro-inflammatory marker expression and increased their anti-inflammatory marker expression on the 7 and 14 days after SCI. The spinal cord H&E staining results showed that EPC-EXOs raised the tissue-sparing area rate significantly after 28 days of SCI and the motor behavior evaluation indicated an increased BMS score and motor-evoked potential by EPC-EXOs treatment after SCI. The RT-qPCR assay identified that miR-222-3P upregulated in EPC-EXOs and its miRNA-mimic also decreased the pro-inflammatory macrophages and increased the anti-inflammatory macrophages. Additionally, miR-222-3P mimic activated the SOCS3/JAK2/STAT3 pathway, and SOCS3/JAK2/STAT3 pathway inhibition blocked miR-2223P’s effects on macrophage polarization and mouse motor behavior.

**Conclusion:**

Comprehensively, we discovered that EPC-EXOs-derived miR-222-3p affected macrophage polarization via SOCS3/JAK2/STAT3 pathway and promoted mouse functional repair after SCI, which reveals EPC-EXOs’ role in modulation of macrophage phenotype and will provide a novel interventional strategy to induce post-SCI recovery.

**Supplementary Information:**

The online version contains supplementary material available at 10.1186/s12974-023-02833-7.

## Introduction

Spinal cord injury (SCI) is a severe traumatic disease of the central nervous system [1] and its pathological mechanism remained unclear, which challenges the development of pro-repair strategies for SCI [2] and thus caused a heavy burden for the whole society [3]. Most studies now consider neuroinflammation as one of the leading causes, and they show that neuroinflammation disabled the axons in the injured area to regenerate the connections between adjacent neurons, resulting in permanent neurological deficits [4–7].

Following spinal cord injury, the released cytokines and chemokines recruit the bone marrow-derived monocytes to the injured area. Then, the monocytes undergo polyphasic differentiation into macrophages and the macrophages impart multiple functions in the damaged environment [8]. Macrophages are mainly divided into pro-inflammatory (M1-like) and anti-inflammatory (M2-like) types as they are polarized to present different functions [9, 10]. The macrophage’s M1-like polarization following spinal cord damage progressively results in a persistent inflammatory state that impedes cell regeneration [11]. In contrast, M2-like macrophages increase cell proliferation and tissue healing. It is advantageous to encourage macrophage polarization toward the anti-inflammatory type in treating CNS damage [12].

Stem/progenitor cell therapy stands out among the therapeutic approaches for spinal cord injury because of its rapid self-renewal, stable multiplication time, and high value-added capacity, thus making it widely used in cell-based therapy and regenerative medicine [13]. Endothelial progenitor cells (EPCs), mainly derived from bone marrow, harbor a multi-differentiation potential and stem cell-specific self-renewal capability. Previous research has shown that bone marrow-derived EPCs migrate to pathologically damaged organs or tissues to promote tissue recovery [14] by encouraging the regrowth of blood vessels and nerve axons [15, 16].

Exosomes are heteroblastic membrane vesicles released by various types of cells into biological fluids and are between 40 and 100 nm in diameter, usually cup-shaped under swept surface electron microscopy [17]. It lacks Golgi apparatus, endoplasmic reticulum, and other organelles that living cells have, but contains proteins (CD9, CD63, Alix, TSG101, and heat shock protein family, etc.), lipids (sphingomyelin, cholesterol, phosphatidylserine, etc.), microRNA, lncRNA, mRNA, rRNA, and other components [18–20]. Exosomes have been widely used in the treatment of spinal cord injury, and exosomes derived from different cells significantly promote the recovery of central nervous function [21–24]. EPC-derived exosomes have been widely researched in lung inflammation [25], sepsis [26], and cardiac injury healing [27], while their roles in SCI remained utterly undiscovered.

Short RNA molecules called microRNAs (miRNAs), which range in size from 19 to 25 nucleotides, control the post-transcriptional silencing of the target genes. One single miRNA can affect the expression of several genes engaged in a functionally interconnected pathway, leading to a considerable transcriptional alteration [28, 29]. Numerous allergic illnesses, such as asthma, eosinophilic esophagitis, allergic rhinitis, and eczema, have been demonstrated to be influenced by miRNAs [30–32]. Recently, multiple investigations suggested that microRNAs may be essential for SCI healing [33, 34]. MiR-222-3p has been identified as a critical factor contributing to CNS injury by preventing neuronal apoptosis [35], encouraging endothelial cell proliferation and migration and therefore mending damage [36], and it stopped macrophages' pro-inflammatory conversion in cancers [37]. Nevertheless, it remains unclear whether miR-222-3p regulates macrophage polarization after SCI.

Suppressor of cytokine signaling 3 (SOCS3) encodes a JAK/STAT axis inhibitor and the SOCS3/JAK2/STAT3 pathway was regulated by miR-222-3p [37]. Besides, SOCS3 has excellent potential to control pro- and anti-inflammatory macrophage polarization [37–42]. Therefore, exosomal miRNA-222-3p derived EPC take its role by regulates the SOCS3/JAK2/STAT3 pathway to affect macrophage polarization, and therefore improve functional recovery after SCI.

## Materials and methods

### Isolation, cell culture, and identification of bone marrow-derived macrophages (BMMs) and endothelial progenitor cells (EPCs)

The primary bone marrow-derived macrophages (BMMs) were extracted as previously documented [43]. Briefly, three C57BL/6 mice were executed via cervical dissection. Under aseptic circumstances, muscle and connective tissue were taken from mouse bones and the extremities of the tibia and fibula. A syringe removes bone marrow and phosphate-buffered saline (PBS) to create a bone marrow stem cells’ suspension. After centrifugation, the cell precipitate was resuspended with BMMs growth medium (IMDM (Gibco, Waltham, MA), 10% FBS (Gibco), 1% antibiotic–antimycotic (Gibco), 10 ng/ml macrophage colony-stimulating factor (M-CSF, PeproTech)). The aforesaid cell suspensions were planted in six-well tissue culture plates, with the initial fluid change on the third day and every other day after that, yielding mature BMMs in roughly a week. Immunofluorescence labeling was used to identify expression markers such as F4/80 and CD68 (Abcam, Cambridge, UK).

Primary bone marrow endothelial progenitor cells (EPCs) were extracted as described previously [44]. Similar to the techniques described above, we acquire bone marrow cell suspensions from C57BL/6 mice, the suspensions were progressively put on top of the Lymphatic Separation Medium (MultiSciences; Hangzhou; China), and mononuclear cells were recovered following centrifugation at 2500*g* × 30 min at 4 °C. They were gently aspirated from the centrifuge tubes and centrifuged at 500 g for 5 min before being resuspended in EPCs growth medium (Endothelial Cell Growth Basal Medium-2 (EBM™-2, LONZA), Endothelial Cell Growth Medium-2 BulletKit™ (EGM™-2, LONZA)). The cell suspension was seeded onto 6-well plates that had already been treated with Human Fibronectin (Sigma), and it was then continuously incubated at 37 °C in a humid environment with 5% CO2. After cell attachment, the media was replaced every two days, and EPCs were collected after around one week. The traditional approach for identifying endothelial progenitor cells uses dual fluorescent labeling (Dil-ac-LDL and FITC-UAE-1 (Solarbio)), and the unique antigens CD133 and VEGFR2 (Cell Signaling Technology, CST) set them apart from regular endothelial cells and other heterogeneous cells.

### Flow cytometry

Endothelial progenitor cells (EPCs) were blocked Fc receptors with mouse CD16/CD32 antibodies (BD) for 15 min at 4 °C in FACS buffer (PBS with 2% FBS and 2 mM EDTA) after cells were digested and harvested, then surface stained with antibodies of CD133 (monoclonal, PE, Biolegend) and VEGFR2 (monoclonal, APC, Biolegend) or isotype control for 30 min at 4 °C. After being washed three times with FACS buffer, cells were resuspended in FACS buffer and run on a BD FACS Canto II (BD). All data were analyzed with Flow Jo (Treestar, OR).

### Isolation of endothelial progenitor cell-derived exosomes (EPC-EXOs) and content analysis

Endothelial progenitor cells were incubated in the complete culture medium with exosome-depleted FBS (160,000 g, 16 h) for 48 h after being grown in the regular medium until 80%–90% confluent. EPC-EXOs were extracted from the finished medium utilizing several techniques, according to the manufacturer's protocol, which can be obtained in one of two ways: either by centrifuging the exosomes in an ultrahigh-speed centrifuge (120000 g, 3 h) or by extracting the exosomes using Amicon^®^ Ultra Centrifugal Filters (Millipore) with ExoQuick-TC for Tissue Culture Media and Urine (System Biosciences, SBI, USA). This is done after the cell supernatant collected and the cell debris removed by preliminary centrifugation (3000*g*, 30 min). The exosomes were dissolved in PBS and kept at − 80 °C for storage. To further describe the morphology of EPC-EXOs, transmission electron microscopy (TEM, FEI company, USA) examination was employed. The diameter and particle count of EPC-EXOs were measured using nanoparticle tracking analysis (NTA, ZetaView, Germany) Immunoblot analysis was used to find the exosome markers CD9, CD63, and TSG101 (Abcam) as well as the cellular marker Calnexin (Abcam).

### Exosomes labeling and uptake assay by BMMs

We labeled the exosomes using a red fluorescent lipophilic dye Dil (Sigma). After being cultured with 100 μg/μl of tagged exosomes for 12 h, BMMS were rinsed with PBS, fixed for 20 min in 4% paraformaldehyde, and stained with Prolong Mounting Media Containing DAPI (GeneTex Inc, Irvine, CA). To ascertain whether exosomes are being picked up, images were acquired using the Zeiss Apotome.

### Spinal cord injury (SCI) model and treatments

According to our earlier work [45], male C57BL/6 wild-type mice were profoundly anesthetized and the spinal cord was moderately contused at the T10 level using a modified Allen weight descender (10 g weight with a vertical height of 20 mm). Following surgery, daily bladder massages were given until full spontaneous or voluntary urination and daily antibiotics were given for three days. For the SCI model consistency, the following criteria were required for inclusion [46]: (I) mice weighing between 20 and 25 g; (II) 10 g weight and 20 mm vertical height requirements for weight reduction devices; (III) tail wagging, hind limb twitching right after SCI, and flaccid paralysis of the hind limbs when awake. Exclusion criteria included: (I) The animals' weight and age were below average. (II) During a laminectomy, the spinal cord unintentionally suffered damage. (III) The mice’s hindlimbs could still move after they emerged from anesthesia. Following SCI, 100 μl of the EPC-EXOs (10 μg/μl) solution was progressively administered into the tail vein, and samples were taken at 7, and 14 days after SCI.

### The tracking tests of EPC-EXOs in vivo

For in vivo tracking tests, EPC-EXOs were tagged with 5 μM of the fluorescent lipophilic dye Dil (Yeasen, Shanghai, China), resuspended in PBS at a concentration of 10 μg/μl, and kept out of the light. Following SCI, the administration of the suspension of Dil-labeled EPC-EXOs was performed via tail vein injection as previously mentioned. Samples were taken seven days after the injury, dehydrated, cut into frozen sections, and examined by immunofluorescence at 594 nm for the absorption of Dil-labeled exosomes by macrophages in the injury region.

### Evaluation of the locomotive behavior function

The BMS (Basso Mouse Scale) method was used to evaluate motor function in the hind limbs before surgery and 1, 3, 7, 14, 21, 28, and 56 days following spinal cord damage. From 0 (totally paralyzed) to 9, the BMS scale measures pain (normal movement). The mean of the BMS primary score and the BMS subscale score was recorded, and the mean value was calculated as the final score value, to prevent bias in the BMS scale data. The BMS subscale system was also combined with the assessment by two researchers who were trained well but had no prior knowledge of the experimental design.

### Footprint analysis

Four weeks after SCI, mice were evaluated for their gait and motor coordination. Different colors were used to paint the front and back paws (red or blue). Groups of mice were put on a straight track with a piece of white paper at the bottom and prompted to go in a straight line. And then we scanned the footprint pattern after being captured in photographs to evaluate the recovery of motor function.

### Neuroelectrophysiology

The mice given effective anesthesia before the motor-evoked potentials (MEPs) were measured. Before surgery (baseline) and 28 days after SCI, the mean MEP values (including amplitude and latency duration) in various intervention groups were obtained. The electrodes were implanted as reported in our prior research [43]. For each mouse, experimental MEP (mV) reflected the relative level of mobility recovery.

### Hematoxylin–eosin staining (HE staining)

After cardiac perfusion 28 days after surgery, mice were sedated with 0.3% pentobarbital, and carefully removed the spinal cord tissue. The samples were first dehydrated with varying levels of alcohol, made transparent with xylene (Macklin), and then embedded in paraffin wax. Once the wax blocks had fully hardened, the samples were divided into sections. The sections were sealed with neutral gum after being stained with hematoxylin and eosin (LEAGENE, Beijing). An N2-Ni-U ortho-fluorescence microscope (Nikon, Shanghai) was used to capture the images.

### Extraction of RNA and quantitative real-time PCR (RT-qPCR) analysis

The miRNA First-Strand cDNA Synthesis Kit 2.0 and miRNA QPCR mix (GeneCopoeia) were used to synthesize cDNA by miRNA reverse transcription and to detect the expression level of the contained miRNAs with a series of miRNA primers (mmu-miR-21a-5p, mmu-miR-222-3p, mmu-miR-c-3p, mmu-miR-155-3p, mmu-miR-29a-3p, mmu-miR-199a-3p, mmu-miR-146a-5p, mmu-let-7i-5p and RNU6) provided by GeneCopoeia, and the above kits contain universal downstream primers. RNU6 was used as an endogenous control to normalize the results.

Bulge-Loop miRNA qRT-PCR Starter Kit, Bulge-Loop miRNA qRT-PCR Primer Set and Bulge-Loop U6 qPCR Primer Set (Ribobio, Guangzhou, China) were used to synthesize cDNA by pri-miR-222 reverse transcription and to detect the expression level of the contained pri-miRNA of macrophages and EPCs-EXOs treated macrophages.

The target genes were reversely transcribed into first-strand cDNA and amplified using the Promega Reverse Transcription System and GoScript™ QPCR Master Mix (Promega) with BRYT Green master mix. The outcomes were calibrated using GAPDH to acquire a more precise reading of the target gene’s expression level. Additional file 1: Table S1 displays the primer sequences.

All reactions run through a real-time PCR system (FTC-3000, Funglyn Biotech Inc., Toronto, Canada) for processing and analysis, and relative gene expressions calculated using the 2 ^−ΔΔCT^ method.

### MiR-222-3p transfection via miR-222-3p mimic, inhibitor, and plasmids

BMMS were seeded in six-well plates, and when their growth was fused to 60%-80%, washed three times with PBS. MiR-222-3p mimic, mimic NC, inhibitor, inhibitor NC (Ribobio, Guangzhou, China) working solution, and Lipofectamine™ 3000 (ThermoFisher) suspension were prepared with Opti-MEM (Gibco), respectively. After standing at room temperature for 10 min, the corresponding suspensions were mixed and then stood at room temperature for another 10 min. BMMs complete medium was added proportionally, the mixed suspensions were added to the cells and the mixed system was reacted in an incubator at 37 degrees for 8 h. The entire medium was then added, and the incubation was maintained for a further 48 h after the cells had been washed three times with PBS. The whole length of the SOCS3 ORF sequence was cloned into the pcDNA 3.1( +) vector using plasmids expressing SOCS3 that were purchased from Changsha Youbio Co, Ltd. Under the manufacturer's instructions, LipofectamineTM 3000 (ThermoFisher) was used to transfect the mimic, inhibitor, and plasmid. The transfected cells were saved for later research.

### Western blot assays

BMMs and spinal cord tissue were lysed using RIPA (Solarbio) with 1% protease and 2% phosphatase inhibitor (Sigma). Protein concentration was measured using a BCA Protein Assay Kit (Solarbio, Beijing, China). And then the proteins were transferred to a polyvinylidene fluoride (PVDF) membrane (Millipore) after being separated using SDS-PAGE gels (Millipore, Billerica, MA). The membrane was incubated with primary antibodies at 4 °C overnight after being blocked for 60 min with 5% non-fat powdered milk (Sangon Biotech) or 5% BSA (Biofroxx) in TBST (Solarbio) at room temperature. The following primary antibodies and dilutions were utilized: rabbit anti-SOCS3 (1:500; Proteintech), rabbit anti-iNOS (1:1000; Proteintech), rabbit anti-Arg-1 (1:5000; Proteintech), rabbit anti-β-actin (1:5000; CST), rabbit anti-JAK2 (1:1000; CST), rabbit anti-p-JAK2 (1:1000; CST), rabbit anti-STAT3 (1:1000; Proteintech), rabbit anti-p-STAT3 (1:1000; CST), rabbit anti-Calnexin (1:1000; Proteintech), rabbit anti-CD9 (1:1000; Abcam), rabbit anti-CD63 (1:1000; Abcam) and rabbit anti-TSG 101 (1:1000; Abcam). The membrane was then treated with peroxidase-conjugated anti-rabbit after being cleaned three times with TBST (1:5000; CST). β-actin was used to evaluate equal protein loadings. The enhanced chemiluminescence reagent (ThermoFisher, Waltham, MA) and ChemiDoc XRS Plus luminescent image analyzer (Bio-Rad) were used to examine the data.

### Immunofluorescence staining

Slices of the spinal cord were permeabilized with 0.3% Triton X-100 in PBS for 30 min and blocked with 5% BSA (Biofroxx) or Animal-Free Blocker (Vectorlabs) for 1 h at room temperature for the spinal cord samples. The cell samples were permeabilized with 0.2% Triton X-100 for 15 min, fixed with 4% PFA in PBS for 20 min, and blocked with 5% BSA for 30 min. Following an overnight incubation at 4 °C, the samples were incubated with the primary antibodies F4/80 (1:100, Santa Cruz), CD68 (1:100, Abcam), FITC-UEA-I (MKbio, USA), Dil-Ac-LDL (MKbio, USA), CD86 (1:200, Abcam), CD206 (1:200, Abcam), VEGFR2 (1:200, CST), CD133 (1:200, Abcam) and Neuronal Class III β-Tubulin (Tuj1) (1:400, Proteintech), followed by incubation with the corresponding secondary antibodies (1:400, Abcam) for 1.5 h at room temperature. TUNEL staining performed according to the kit manual (Elabscience). The slices were mounted using Prolong Mounting Media Containing DAPI after being cleaned three times with PBS (GeneTex Inc, Irvine, CA). Using the Zeiss Apotome, variations in the fluorescence intensities of F4/80, CD86, and CD206 were computed for the quantitative investigation of the pro-inflammatory and anti-inflammatory phenotypes. To measure the area of F4/80^+^ cells, four identically sized areas were randomly chosen from the injury center. In each area, the green fluorescent area in response to macrophage aggregation was measured in each group, and the value of this area was used to calculate the area of F4/80^+^ cells. Calculating the number of F4/80^+^CD86^+^ cells or the proportion of F4/80^+^CD206^+^ cells to F4/80^+^ cells in an area of a certain size allowed researchers to study the polarization of macrophages (Image-Pro Plus 6.0).

### Luciferase reporter assay

24-well plates were planted with HEK 293 T cells. *Mus musculus* wild-type (WT) or mutant (MUT) SOCS3 3′UTR segments were cloned into the pmiR-RB-Report™ plasmid vector for the creation of plasmids (Ribobio, Guangzhou, China). After that, HEK 293 T cells were transfected with the plasmids (miR-222-3p mimic, inhibitor, mimic NC, and inhibitor NC). After 48 h, a 1 × PLB cell lysis solution was added, and the cells were agitated for 15 min at room temperature. Cell scrapers were then used to collect the cell lysate. A total of 20 μl of cell lysate and 100 μl of each of the following solutions were applied to each well of a 96-well plate: 100 μl of Luciferase Assay Buffer II and 100 mL of Stop & Glo^®^ Substrate, respectively. We accomplished this experiment using the Promega Dual-Luciferase system (Promega, USA). The firefly luciferase and Renilla luciferase activity were detected using the Centro XS3 LB 960 (Berthold, Germany) and MikroWin software, and their differences were quantified and examined.

### Statistical assay

Software for statistical analysis included GraphPad Prism 8.0 (La Jolla, CA, USA) and SPSS 22.0 (SPSS, Inc.). All data displayed as means ± standard error of measurement (SEM). Researchers who carried out statistical analysis were treated in a blind manner. Two groups were compared using the unpaired *t*-test, and pre- and post-treatment data were compared using the paired t-test. One-way or two-way ANOVA was used with Tukey's post hoc test to analyze the differences between three or more groups or between the groups over time. Statistical significance was defined as p less than 0.05.

## Results

### Bone marrow-derived macrophages (BMMs), endothelial progenitor cells (EPCs), and exosomes (EPC-EXOs) extraction and identification

**Fig. 1:**
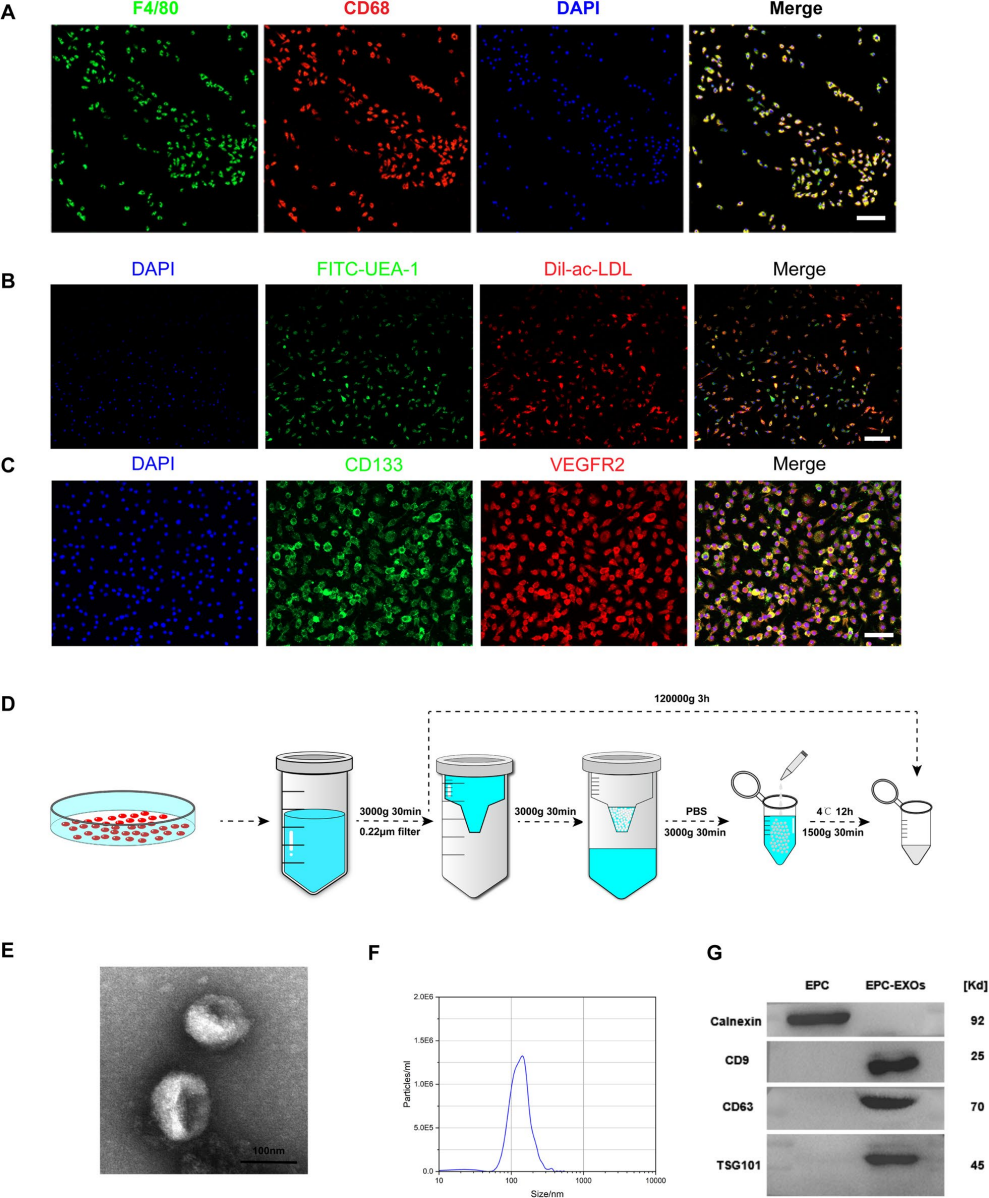
Bone marrow-derived macrophages (BMMs), endothelial progenitor cells (EPCs), and exosomes (EPC-EXOs) extraction and identification. **A** Immunocytochemical identification of primary mice bone marrow macrophages. Scale bar: 100 µm. **B** The bone marrow-derived EPCs were further defined by their ability to bind FITC-UEA-l and uptake Dil-ac-LDL. Scale bar: 100 μm. **C** Immunostaining revealed that the adherent cells were positive for CD133 and VEGFR2. Scale bar: 50 μm. **D** A schematic diagram of the extraction of EPC-EXOs. **E** Transmission electron microscopy (TEM) image of EPC-EXOs. Scale bar: 100 nm. **F** Nanoparticle tracking analysis (NTA) of EPC-EXOs diameter and particle number. **G** Immunoblotting of cell marker Calnexin and exosomal surface markers CD63, CD9, and TSG101

We first obtained BMMS and EPCs from femoral and tibial bone marrow of C57BL/6 mice. Then, we observed the morphology of BMMs and EPCs under a microscope, and Additional file 1: Fig. S1 shows that the endothelial progenitor cells grew in typical colonies at an early stage and the cell morphology resembled that of endothelial cells. Furthermore, the isolated cells highly expressed mature macrophage surface markers F4/80 and CD68 (Fig. 1A). The monocytes were successfully induced into EPCs as the classical EPC identifiers FITC-UEA-1 and Dil-ac-LDL were detected (Fig. 1B). Also, they highly expressed specific stem and progenitor cell markers (CD133) and vascular growth factor receptor 2 (VEGFR2) as exhibited in Fig. 1C. In addition, the results of flow cytometry experiments (Additional file 1: Fig. S2) demonstrated that the extracted cells highly expressed endothelial progenitor cell surface markers (CD133 and VEGFR2). The above results showed that we have successfully obtained BMMS and EPCs.

Next, we collected the supernatant of EPCs and isolated the exosomes by ultra-high-speed centrifugation and the kit method (Fig. 1D). Next, we isolated the exosomes from the EPC supernatants and used TEM, NTA, and western blots to verify their characteristic. The TEM images presented that exosomes possessed a typical double-layer membrane structure (Fig. 1E) and the NTA results showed the diameter of the exosomes between 30 and 150 nm (Fig. 1F). Additionally, the outcomes of the western blot revealed that the surface markers of cells (Calnexin) and exosomes (CD9, CD63, and CD81) were both expressed (Fig. 1G). Overall, these results showed that we successfully induced the bone marrow-derived monocytes into EPCs and BMMS and obtained the EPC-EXOs.

### EPC-EXOs can be taken up by BMMs and promote anti-inflammatory macrophages in vitro

**Fig. 2:**
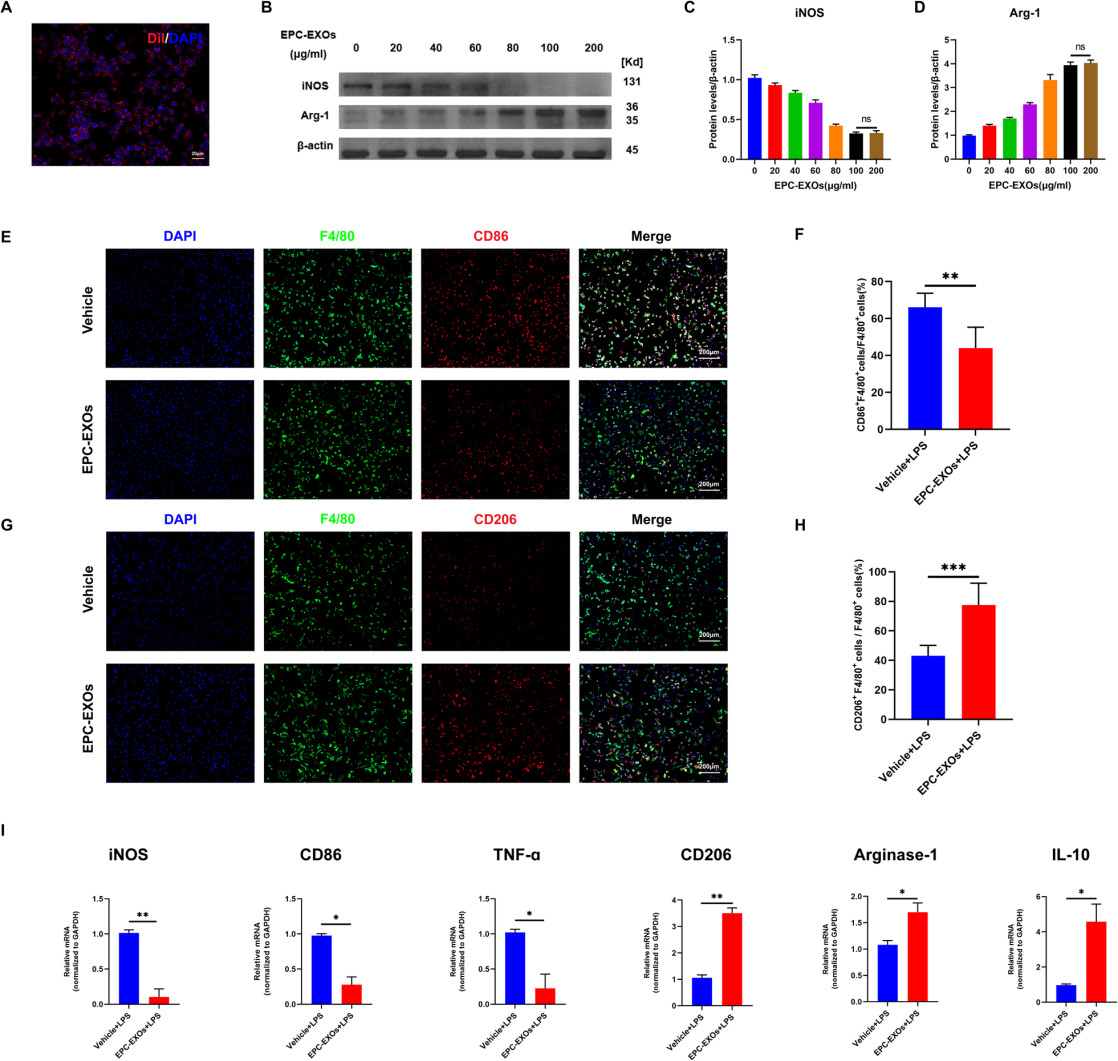
EPC-EXOs can be taken up by BMMs and promote anti-inflammatory macrophages in vitro. **A** Representative Dil-labeled EPC-EXOs in BMMs. Red fluorescence indicates Dil-labeled EPC-EXOs and blue fluorescence indicate BMMs’ nucleus. Scale bar: 20 µm. **B** Western Blotting analysis of the expression levels of iNOS and Arg-1 after LPS stimulation of macrophages with different concentration gradients of EPC-EXOs. **C** Statistical analysis of the expression levels of iNOS. ^ns^P > 0.05 vs 100ug/ml EPC-EXOs group. **D** Statistical analysis of the expression of levels of Arg-1. ^ns^P > 0.05 vs 100ug/ml EPC-EXOs group **E** Representative immunofluorescence images of Vehicle and EPC-EXOs interfering with CD86 (red) and DAPI (blue) staining of F4/80^+^ macrophages (green). Scale bar: 200 μm. **F** Quantification of CD86^+^ cells in each group, *n* = 3 per group, **P < 0.01. **G** Representative immunofluorescence images of Vehicle and EPC-EXOs interfering with CD206 (red) and DAPI (blue) staining of F4/80^+^ macrophages (green). **H** Quantification of CD206-positive cells in each group. *n* = 3, **P < 0.01. **I** qRT-PCR analysis of mRNA expression of pro-inflammatory macrophages’ markers (iNOS, CD86 and TNF-α) and anti-inflammatory macrophages’ markers (CD206, Arginase-1 and IL-10) in macrophages exposed to LPS by Vehicle and EPC-EXOs intervention. *n* = 6, *P < 0.05, **P < 0.01

Before co-culturing BMMs with EPC-EXOs for 24 h, we tagged the EPC-EXOs with the red lipophilic fluorescent dye Dil and the confocal images exhibited the up-taken EPC-EXOs by BMMs (Fig. 2A).

We opted to produce BMMs with LPS to imitate the injury scenario in vivo since prior research [47] has shown that macrophages attracted to the wounded region following SCI are mostly oriented toward pro-inflammatory. We co-cultured the BMMS with gradient EPC-EXOs concentrations of 0, 20 μg/ml, 40 μg/ml, 60 μg/ml, 80 μg/ml, 100 μg/ml, and 200 μg/ml to select an optimal EPC-EXOs concentration to affect BMMs polarization, and we used Western blotting to detect the pro-inflammatory macrophages’ marker iNOS and the anti-inflammatory macrophages’ marker Arg-1 expression. As a result, no statistically significant differences in iNOS and Arg-1 levels between the 100 µg/ml and the 200ug/ml treated group were observed (Fig. 2B–D), and the exosome concentration of 100ug/ml was chosen for the subsequent in vitro experiments. Immunofluorescence results (Fig. 2E–H) demonstrated that the fluorescence intensity of CD86^+^F4/80^+^ (proinflammatory) was significantly diminished in BMMs treated with EPC-EXOs compared to the Vehicle group, whereas the fluorescence intensity of CD206^+^F4/80^+^ (anti-inflammatory macrophages’ marker) was significantly increased. This further demonstrated that EPC-EXOs can promote macrophage anti-inflammatory polarization. Similar results were obtained by the RT-qPCR data (Fig. 2I) as M2 polarization markers expression was upregulated after EPC-EXOs treatment.

### EPC-EXOs are taken up by macrophages in the injured spinal cord region and promote macrophages' anti-inflammatory polarization

**Fig. 3:**
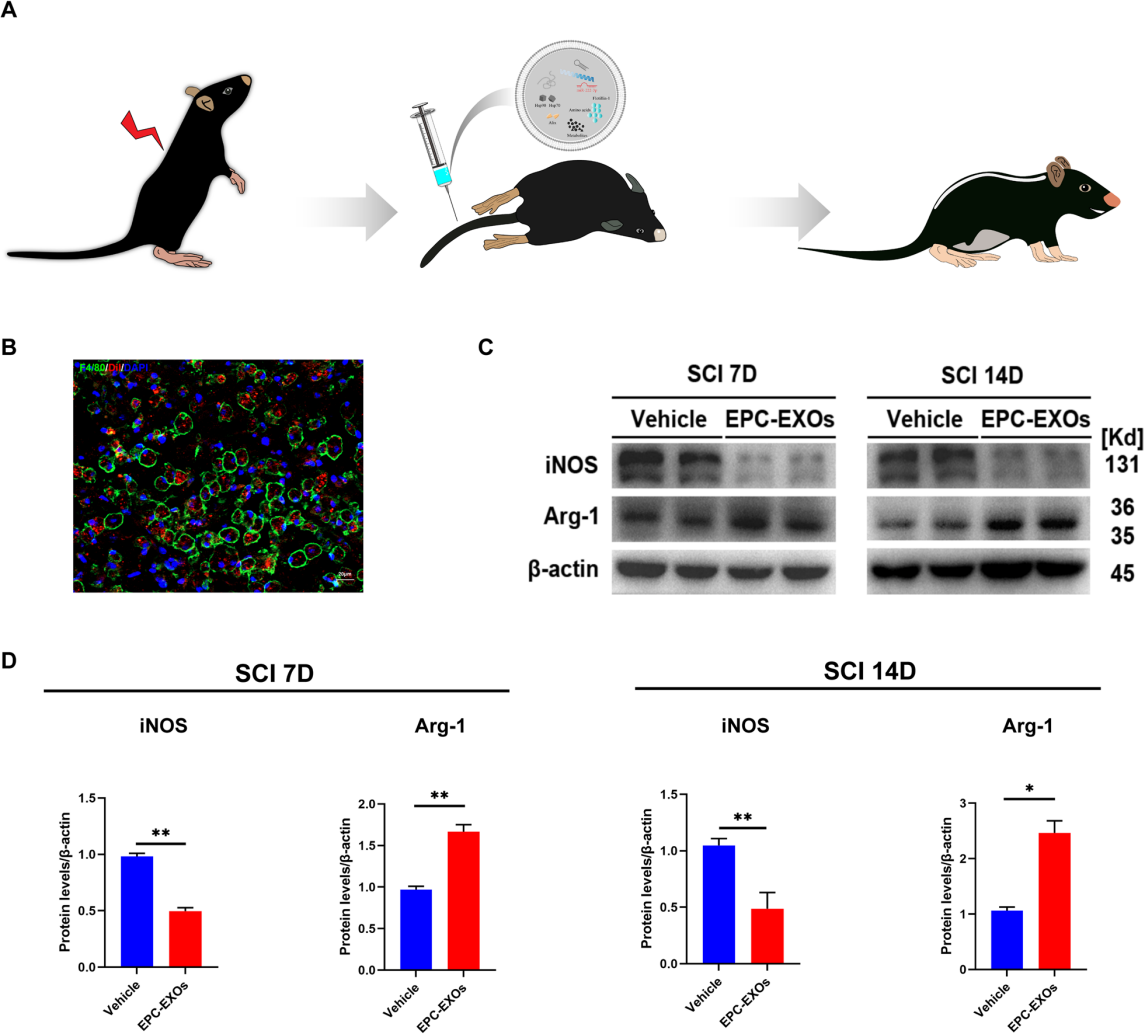

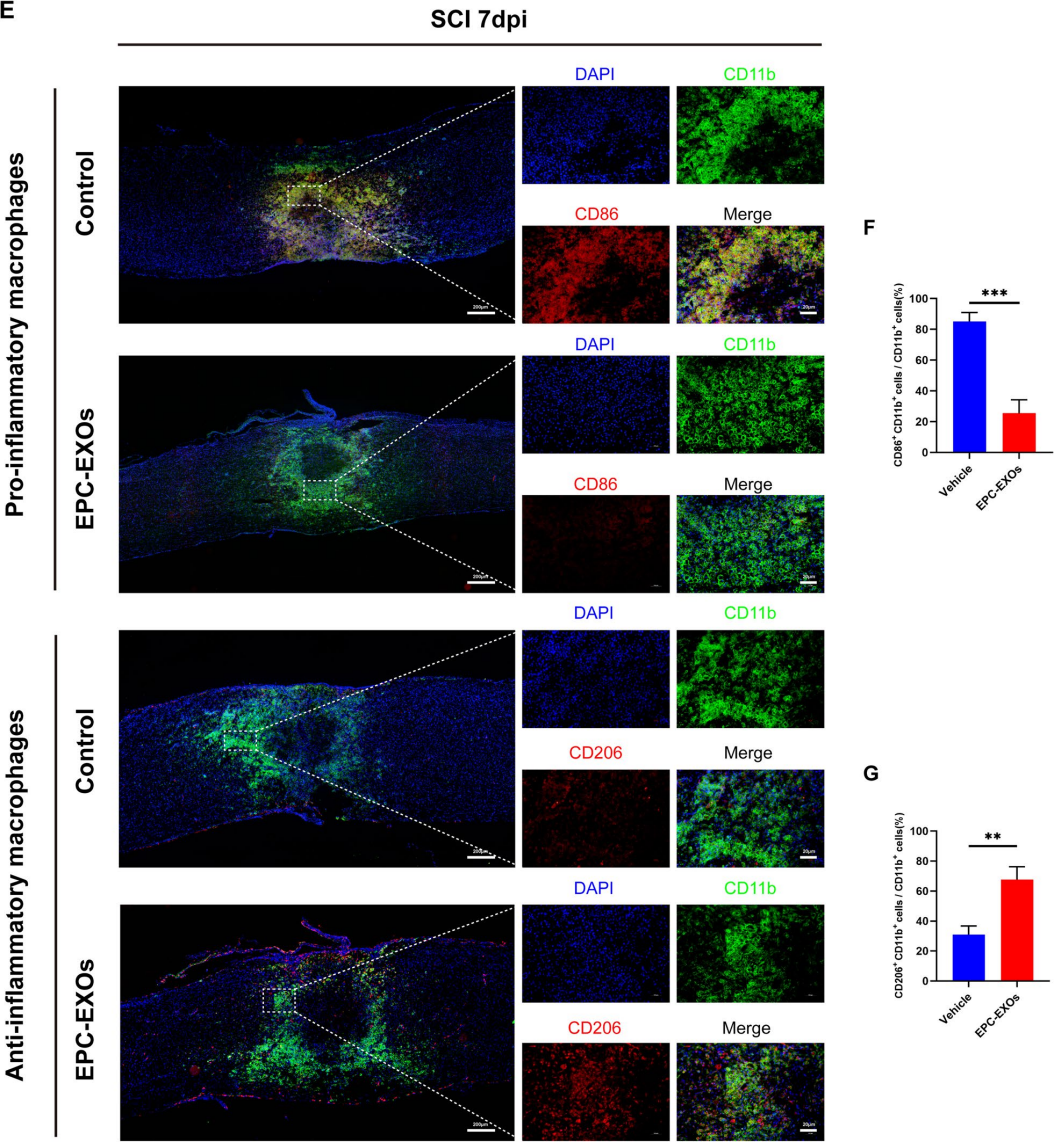

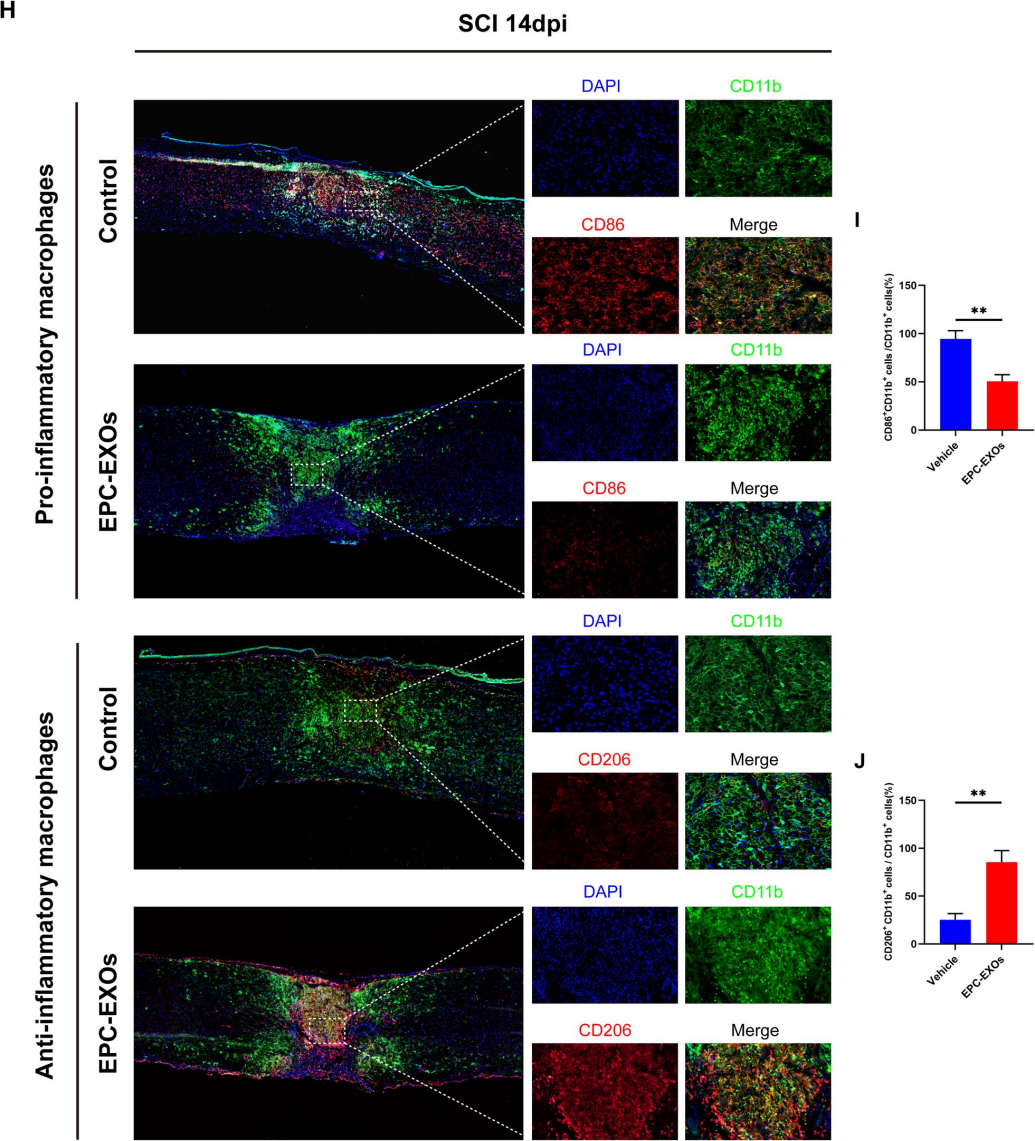

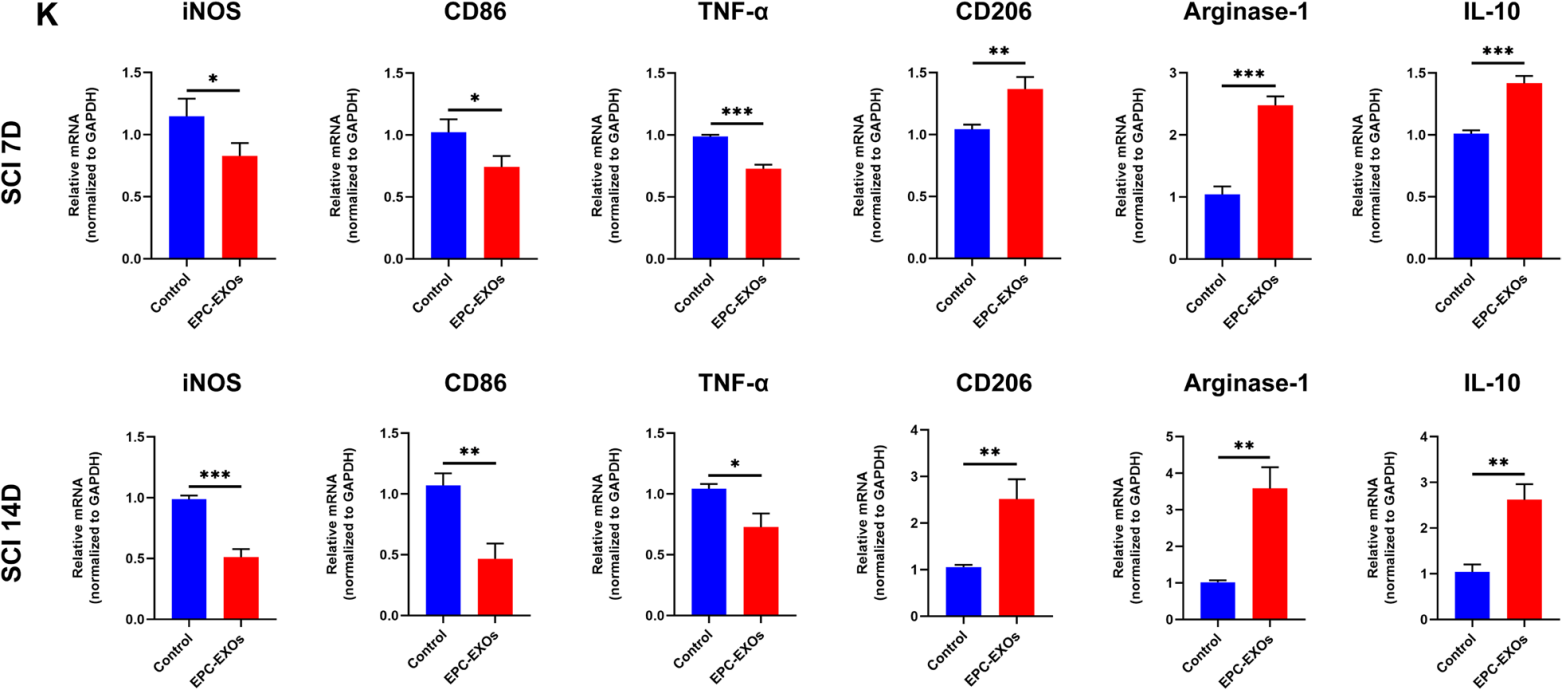
EPC-EXOs are taken up by macrophages in the injured spinal cord region and promote macrophages’ anti-inflammatory polarization. **A** Diagrammatic representation of the tail vein injection of EPC-EXOs (200 μg). **B** Representative images of EPC-EXOs internalized by BMMs. Scale bar: 20 μm. **C** Western Blotting detected changes in the expression levels of iNOS and Arg-1 in macrophages in the region of injury at 7 and 14 days of spinal cord injury in Vehicle and EPC-EXOs-treated wild-type mice. **D** Statistical analysis of the expression levels of iNOS and Arg-1, *n* = 3, *P < 0.05, **P < 0.01. **E** Immunofluorescence images of CD11b^+^ macrophages (green), CD86^+^ (M1) /CD206^+^ (M2) (red) and DAPI (blue) staining of spinal cord injury sections of mice representative of the Vehicle group and EPC-EXOs group at 7 days post-injury (dpi) in wild-type mice. **F**, **G** Immunofluorescence results were analyzed by ImageJ, GraphPad, and SPSS, *n* = 3, **P < 0.01. **H** Immunofluorescence images of CD11b^+^ macrophages (green), CD86^+^ (M1) /CD206^+^ (M2) (red) and DAPI (blue) staining of spinal cord injury sections of mice representative of the Vehicle group and EPC-EXOs group at 14 days post-injury (dpi) in wild-type mice. **I**, **J** Immunofluorescence results were analyzed by ImageJ, GraphPad, and SPSS. *n* = 3, **P < 0.01. **K** qRT-PCR analysis of mRNA expression of pro-inflammatory markers (iNOS, CD86 and TNF-α) and anti-inflammatory markers (CD206, Arginase-1 and IL-10) in macrophages in the region of injury at 7 and 14 dpi in Vehicle and EPC-EXOs-treated wild-type mice, *n* = 6, *P < 0.05, **P < 0.01, ***P < 0.001

To further clarify the effect of EPC-EXOs on macrophage polarization in vivo, we established a spinal cord injury model using wild-type (WT) mice at 6–8 weeks, divided into Vehicle and EPC-EXOs groups, with Vehicle groups as the control. We administered Dil-labeled-EXOs (200 μg/mouse), unlabeled EPC-EXOs (200 μg/mouse), and PBS via tail vein injection to mice with spinal cord injury (Fig. 3A). Exosomes injected into the tail vein can cross the compromised blood–spinal cord barrier to reach the affected location and perform therapeutic effects. Dil-labeled-EXOS was phagocytosed by F4/80^+^ macrophages in the injury location, as shown in Fig. 3B, and then accumulated in the cytoplasm. By counting, 80% of the F4/80^+^ cell population phagocytosed the Dil-labeled EPC-EXOs in the damaged area, i.e., proving that most of our injected EPC-EXOs were taken up by macrophages and exerted subsequent effects (Additional file 1: Fig. S3). We selected the follow-up trials on days 7 and 14 following the SCI as optimal time points to observe macrophage polarization as previous studies suggested [48, 49], and the fresh proteins were collected for Western Blotting. According to Fig. 3C, D, Arg-1 was upregulated in the EPC-EXOs group on both days 7 and 14, indicating the activated anti-inflammatory polarization of macrophages induced by EPC-EXOs. Using immunofluorescence staining, similar results were obtained as the ratio of CD206^+^ F4/80^+^ macrophages to the total number of F4/80^+^ cells was significantly higher in the EPC-EXOs group compared to the Control group, and the ratio of CD86^+^ F4/80^+^ macrophages was significantly lower in the EPC-EXOs group, at both days 7 (Fig. 3E–G) and 14 (Fig. 3H–J) after SCI. The further RT-qPCR results (Fig. 3K) also accorded with the results above as the expression of M1-type markers (iNOS, CD86, and TNF-) by macrophages in the EPC-EXOs group was considerably lower and the expression of anti-inflammatory macrophages’ markers (CD206, Arginase-1, and IL-10) was significantly higher, at both day 7 and day 14 following SCI. Shortly, we found that EPC-EXO encourages macrophage polarization toward the anti-inflammatory phenotype.

### EPC-EXOs attenuate tissue damage and improve functional recovery post-injury

**Fig. 4:**
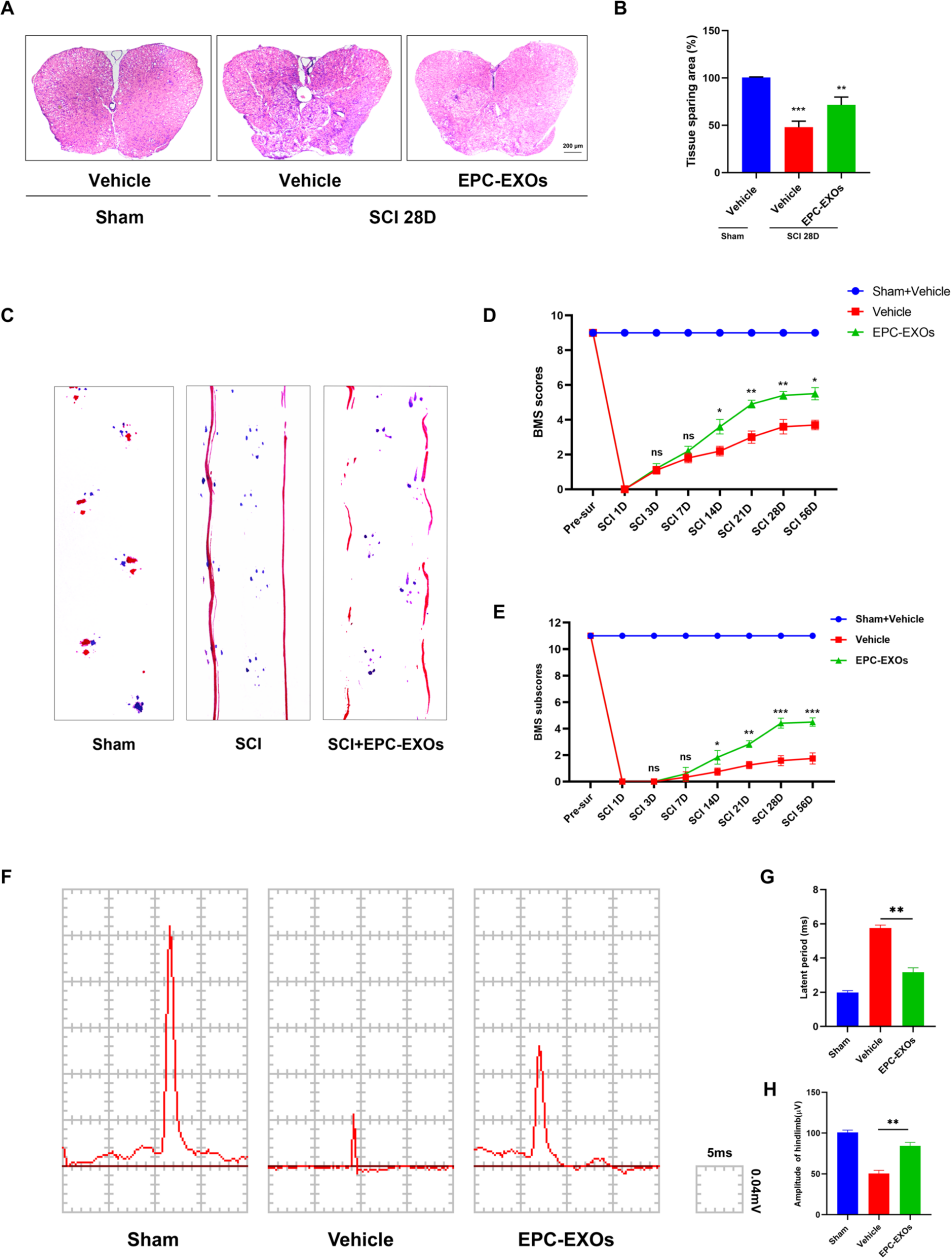
EPC-EXOs attenuate tissue damage and improve functional recovery post-injury. **A** H&E staining of Sham, Vehicle, and EPC-EXOs group at SCI 28 days. Scale bar: 200 μm. **B** Quantification of **A**, *n* = 3, **P < 0.01, ***P < 0.001. **C** Representative footprints of animal walking 28 days post-SCI. Blue: front paw print; red: hind paw print. **D**, **E** BMS primary score and the BMS subscale score over time post-SCI in Sham, Vehicle and EPC-EXOs group, *n* = 5, ^ns^P > 0.05, *P < 0.05, **P < 0.01, ***P < 0.001. **F** Representative images of motor-evoked potential (MEP) in Sham, Vehicle, and EPC-EXOs group at 28 days post-SCI. *n* = 6. **G**, **H** Quantification of **F**, *n* = 6, **P < 0.01

The histological examination and behavior tests were carried out to further determine whether EPC-EXOs can facilitate the restoration of neurological function and tissue healing. After H&E staining, we found that the wounded area dramatically decreased after EPC-EXOs therapy (Fig. 4A, B). Then, we used footprint analysis (Fig. 4C) to gather gait information from the EPC-EXOs, SCI, and sham groups. After SCI, the motor function of the mouse hind paw was degraded compared to the sham group, and the coordination of front and rear paw movements was greatly reduced; however, the motor function and coordination were dramatically improved after EPC-EXOs treatment. Furthermore, BMS scores and sub-scores indicated that motor function was fully lost in the Vehicle and EPC-EXOs groups on the first day and began to recover after 3 days, although there was no statistically significant difference until day 7. After spinal cord injury, the EPC-EXOs group considerably outperformed the Vehicle group in terms of BMS scores and sub-scores at 14D, 21D, 28D, and 56D (Fig. 4D, E). These findings imply that following spinal cord damage, EPC-EXOs may aid in the restoration of motor function in the hind limbs.

Additionally, the electrophysiological research showed that the injection of EPC-EXOs resulted in much shorter latent periods and a much larger amplitude of motor-evoked potentials (MEP) at 28 days post-SCI (Fig. 4F–H). To determine whether EPC-EXOs benefit axonal regrowth at the microscopic level, we further stained the mice's spinal cords 28 days after spinal cord injury. Axons at both ends of the injured core grew back into the injured area, as shown in the immunofluorescence staining images (Additional file 1: Fig. S4A), indicating that EPC-EXOs significantly promoted axonal regeneration, which was in contrast to the vehicle group. Additionally, we noticed that the presence of EPC-EXOs substantially decreased the amount of apoptosis in the region of injury (Additional file 1: Fig. S4B, C). The above findings imply that EPC-EXOs greatly improve the restoration of motor function and neurophysiological activity in the hind limbs following SCI.

### EPC-EXOs-derived miR-222-3p promotes anti-inflammatory macrophages

**Fig. 5:**
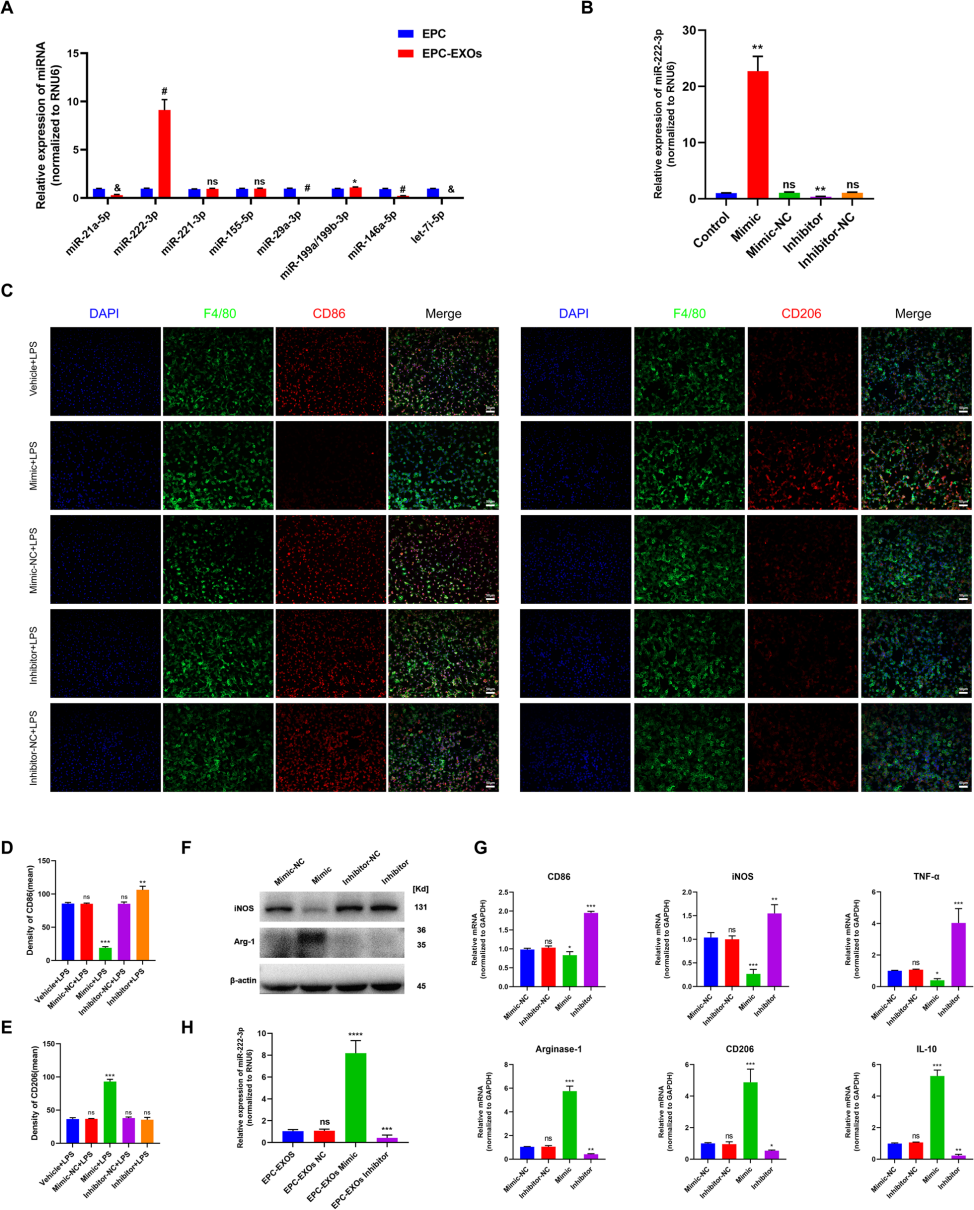

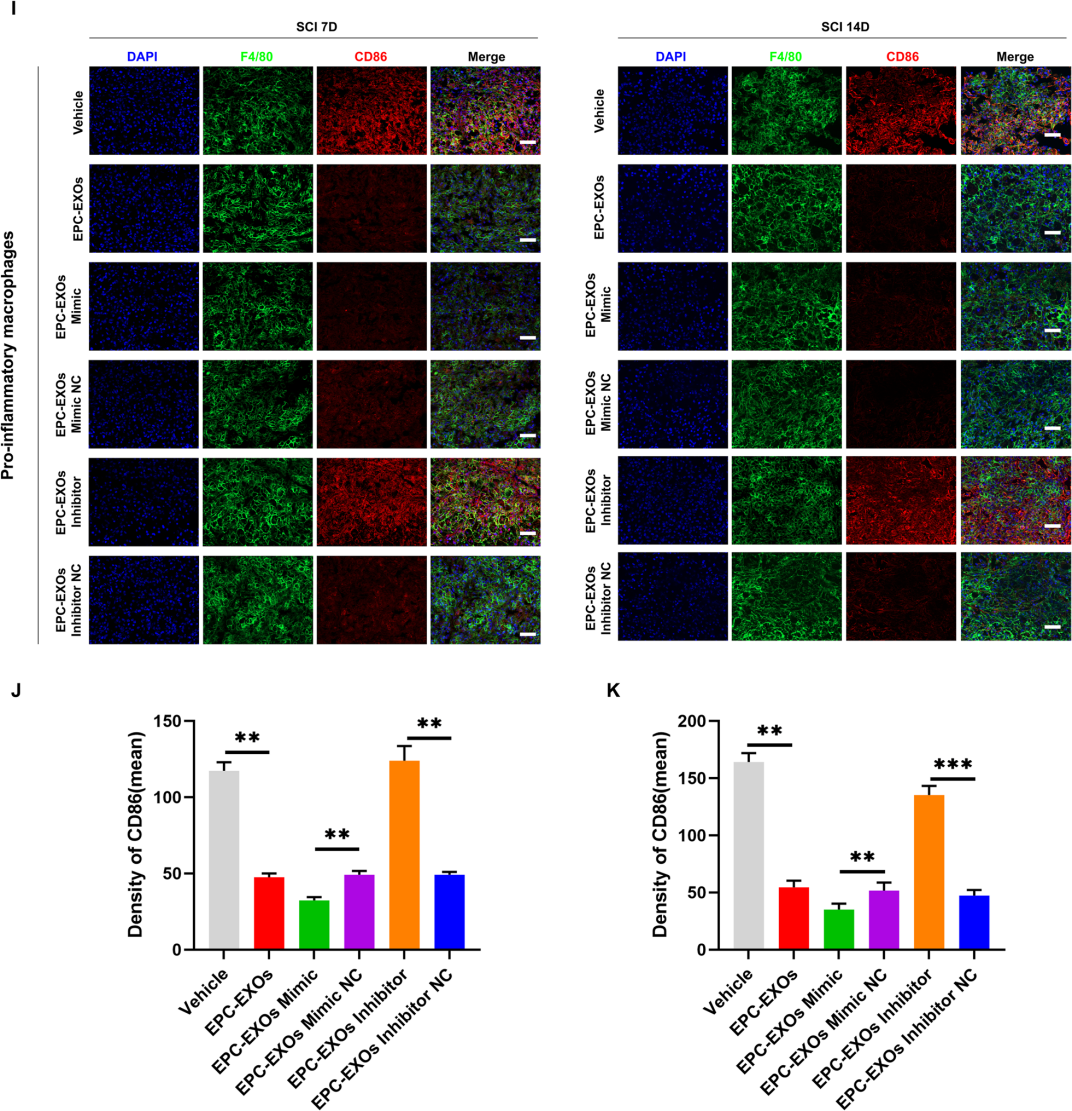

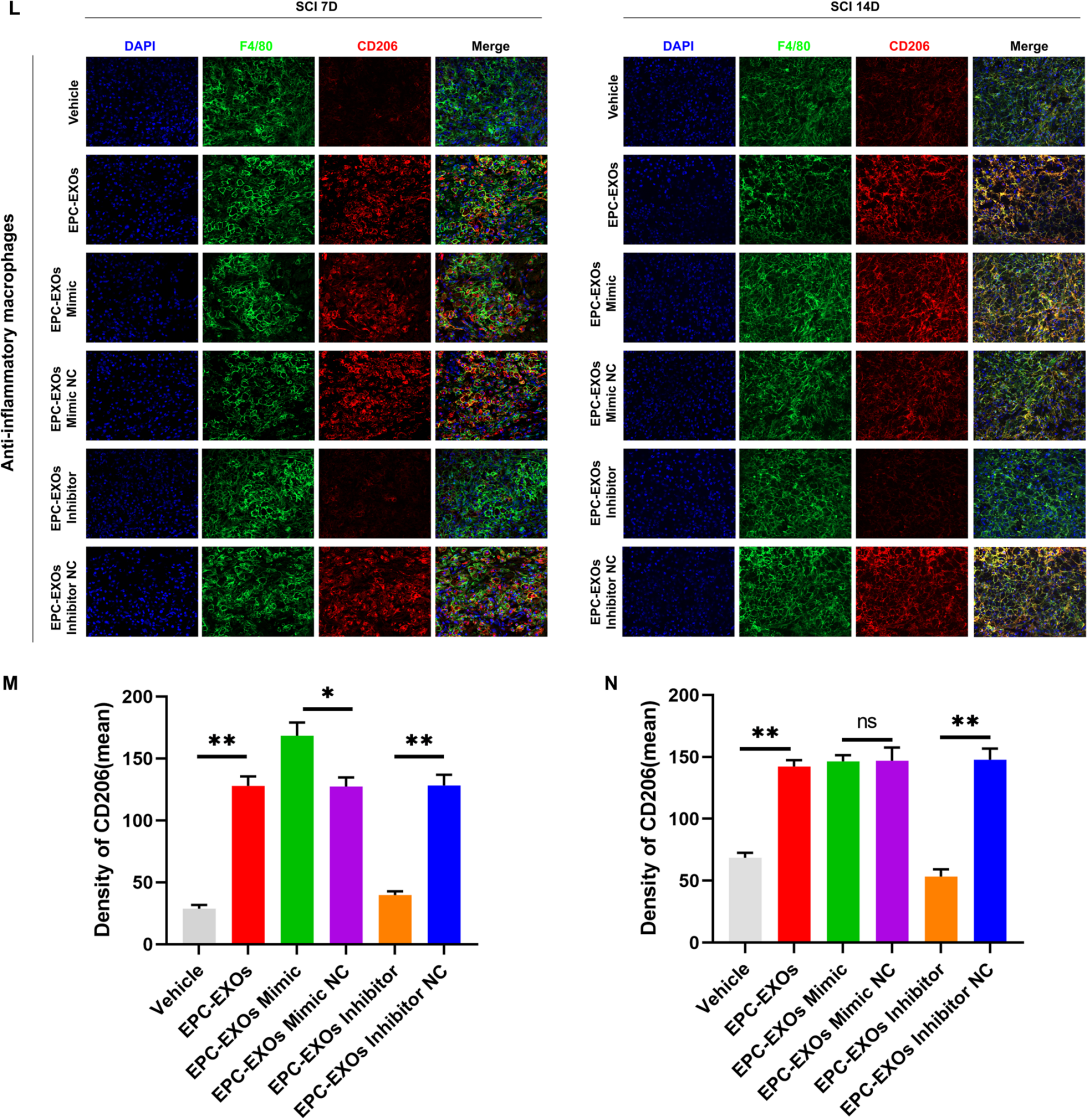

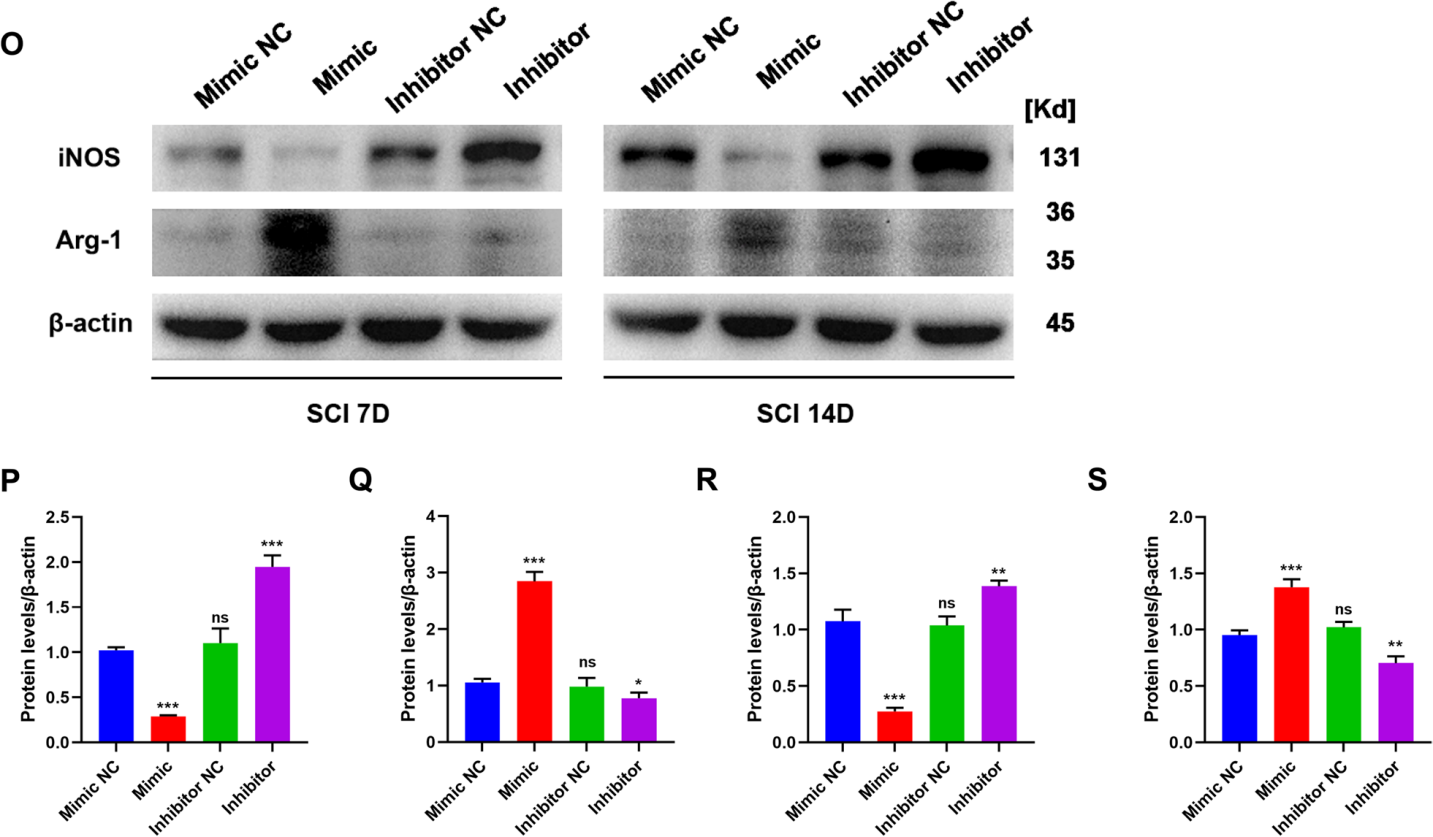
EPC-EXOs-derived miR-222-3p promotes anti-inflammatory macrophages. **A** The relative expression of candidate genes was detected by qRT-PCR in EPCs and EPC-EXOs, *n* = 6, ^ns^*P* > 0.05, *P < 0.05, ^&^*P* < 0.001, ^#^*P* < 0.0001. **B** The transfection efficiency of miR-222-3p mimic/mimic NC/inhibitor/inhibitor NC in BMMs was detected by qRT-PCR, *n* = 3, ^ns^*P* > 0.05, *P < 0.05, **P < 0.01. **C** Representative immunofluorescence images of the groups: Vehicle + LPS, Mimic + LPS, Mimic NC + LPS, Inhibitor + LPS and Inhibitor NC + LPS interfering with CD86/ CD206 (red) and DAPI (blue) staining of F4/80^+^ macrophages (green). Scale bar: 50 μm. **D, E** Quantification of CD86^+^ or CD206^+^ cells in each group, *n* = 3, ^ns^*P* > 0.05, **P < 0.01, ***P < 0,001. **F** Western blotting analysis of the expression levels of iNOS (pro-macrophage macrophages’ marker) and Arg-1 (anti-inflammatory macrophages’ marker) after LPS stimulation of macrophages with Mimic, Mimic NC, Inhibitor and Inhibitor NC. **G** qRT-PCR analysis of mRNA expression of pro-macrophage macrophages’ markers (iNOS, CD86 and TNF-α) and anti-inflammatory macrophages’ markers (CD206, Arginase-1 and IL-10) in macrophages treated with miR-222-3p-Mimic/Mimic NC/Inhibitor/Inhibitor NC, *n* = 3, *P < 0.05, **P < 0.01, ***P < 0.001. **H** The transfection efficiency of miR-222-3p mimic, inhibitor and NC in EPC-EXOs was detected by qRT-PCR, *n* = 3, ***P < 0.001, ****P < 0.0001. **I**, **L** Immunofluorescence images of F4/80^+^ macrophages (green), CD86^+^/CD206^+^ (red) and DAPI (blue) staining of spinal cord injury sections of mice representative of the groups: Vehicle, EPC-EXOs, EPC-EXOs Mimic, EPC-EXOs Mimic NC, EPC-EXOs Inhibitor, and EPC-EXOs Inhibitor NC at 7- and 14- days post-injury (dpi) in wild-type mice. Scale bar: 50 μm. **J**–**N** Immunofluorescence results were analyzed by ImageJ, GraphPad, and SPSS. *n* = 3, ^ns^*P* > 0.05, *P < 0.05, **P < 0.01, ***P < 0.001. **O** Western Blotting detected changes in the expression levels of iNOS and Arg-1 in macrophages in the region of injury at 7 and 14 days of spinal cord injury in the groups: EPC-EXOs-Mimic NC, Mimic, Inhibitor NC and Inhibitor-treated wild-type mice. **P**–**S** Statistical analysis of the expression levels of iNOS and Arg-1, *n* = 3, ^ns^P > 0.05, *P < 0.05, **P < 0.01, ***P < 0.001

Exosomes comprise lipids, proteins, miRNAs, mRNAs, circular RNAs, and DNA produced from cells. These exosomes include mRNAs and miRNAs that continue to function after entering recipient cells, such as translating proteins [50, 51]. Previous research has used next-generation sequencing to examine the miRNA components in EPC-EXOs and screened for the most highly expressed miRNAs [44]. We applied RT-qPCR and found that the miR-222-3p expression was higher in the EPC-EXOs group compared to the EPC group (Fig. 5A), demonstrating that miR-222-3p was exceptionally abundant in EPC-EXOs. Exosome-borne miRNAs are exogenous miRNAs, and their alteration of BMMs should only influence the mature body they carry; related miRNA precursors should remain unaffected. We created pri-miRNA primers to verify this theory, and through the use of qRT-PCR, we discovered that the pri-miRNAs in BMMs did not significantly alter as a result of exosome intervention (Additional file 1: Fig. S5). Utilizing immunofluorescence labeling, Western Blotting, and RT-qPCR on macrophage polarization following lipopolysaccharide (LPS) stimulation, we further validated the role of miR-222-3p in mediating macrophage polarization. Subsequently, we manipulate the miR-222-3p expression by miR-mimic and inhibitor. As exhibited in Fig. 5B, the expression level of miR-222-3p was significantly higher in the mimic group when compared to the control group, while it was significantly lower in the inhibitor group. Immunofluorescence staining (Fig. 5C–E) revealed that the mean CD86 fluorescence intensity was substantially more down in the macrophages treated with miR-mimic, whereas the mean CD206 fluorescence intensity was much higher. Besides, the CD86 expression upregulated after inhibitor treatment. Similar results were obtained by western blotting and RT-qPCR (Fig. 5F, G) that the mimic group presented a substantial decrease in the expression of iNOS (pro-inflammatory macrophages’ marker) and a significant increase in the expression of Arg-1 (anti-inflammatory macrophages’ marker). Also, the mRNA expression of the pro-inflammatory markers CD86 and TNF-α decreased, and anti-inflammatory associated markers CD206 and IL-10 increased in the mimic group.

We initially transfected EPCs and then collected their exosomes in order to further evaluate the impact of miR-222-3p on macrophage polarization in the regional center of spinal cord injury in vivo. We next used RT-qPCR to assess the transfection effectiveness (Fig. 5H). It was evident that miR-222-3p was considerably more abundant in exosomes after mimic transfection compared to the EPC-EXOs group, whereas inhibitor had the opposite effect. Following that, we gave wild-type C57BL/6 mice with spinal cord injuries transfected EPC-EXOs by tail vein injection, and we took samples on days seven and fourteen after the injury. We stained the frozen sections with immunofluorescence, and as shown in Fig. 5I–K, on either the seventh- or fourteenth-day following injury, the mean fluorescence intensity of CD86 was significantly lower in the EPC-EXOs Mimic group compared to the NC group, with the Inhibitor group exhibiting the opposite pattern. The EPC-EXOs Mimic and Inhibitor groups displayed the expected results for CD206 at day seven after injury, as shown in Fig. 5L–N. However, at day fourteen after injury, the EPC-EXOs Mimic group did not show a statistically significant difference compared to the NC group, possibly confirming the earlier findings that macrophages started to polarize significantly toward anti-inflammation at day fourteen after SCI. Additionally, the outcomes of Western blotting provided solid support for our initial hypotheses. At days seven and fourteen following injury, as seen in Fig. 5O–S, the expression level of Arg-1 in the Mimic group was dramatically increased and decreased in the inhibitor group. Reversely, iNOS showed the opposite expression trend. In summary, miR-222-3p loaded by EPC-EXOs effectively encouraged the development of anti-inflammatory macrophages while inhibiting the polarization of macrophages toward pro-inflammatory macrophages both in vivo and in vitro.

### MiR-222-3p promotes anti-inflammatory macrophages via the SOCS3/JAK2/STAT3 signaling pathway

**Fig. 6:**
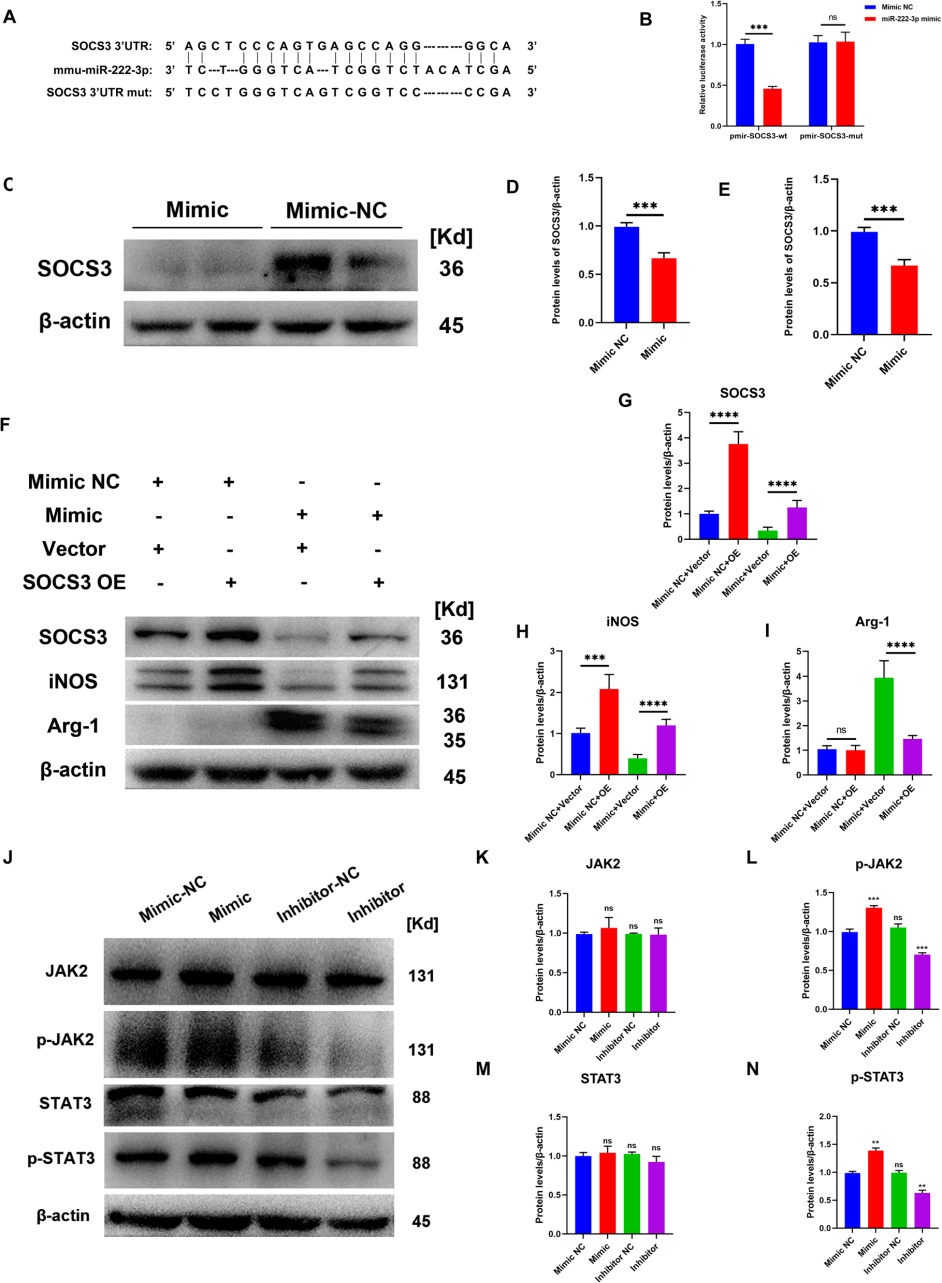
MiR-222-3p promotes anti-inflammatory macrophages via the SOCS3/JAK2/STAT3 signaling pathway. **A** Schematic diagram of miR-222-3p and SOCS3′s 3′UTR binding sites. **B** Dual-luciferase reporter assays were performed to determine the targeting sequence of miR-222-3p and 3′UTR of SOCS3. **C** Western Blotting analysis of the expression levels of SOCS3 in macrophages with miR-222-3p-Mimic and Mimic NC. **D** Statistical analysis of the expression levels of SOCS3, *n* = 3, ***P < 0.001. **E** qRT-PCR analysis of mRNA expression of SOCS3 in macrophages with miR-222-3p-Mimic, Mimic NC, Inhibitor and Inhibitor NC, *n* = 3, ^ns^P > 0.05, **P < 0.01. **F** Western blot assay was used to detect expression levels of SOCS3, iNOS and Arg-1 after co-transfection. **G**–**I** Statistical analysis of the expression levels of SOCS3, iNOS and Arg-1, *n* = 3, ***P < 0.001, ****P < 0.0001. **J** Western Blotting analysis of the expression levels of JAK2, p-JAK2, STAT3 and p-STAT3 in macrophages with miR-222-3p-Mimic, Mimic NC, Inhibitor and Inhibitor NC. **K**–**N** Statistical analysis of the expression levels of JAK2, p-JAK2, STAT3 and p-STAT3, *n* = 3, ^ns^P > 0.05, *P < 0.05, **P < 0.01, ***P < 0.001

To understand the underlying mechanism of miR-222-3p-mediated macrophage polarization, we investigated the putative target genes of miR-222-3p. As SOCS3 is a potential target gene of miR-222-3p and regulates inflammation, we estimated the potential binding between miR-222-3p and SOCS3. We noticed that the corresponding sequence of miR-222-3p combines in the 3′ untranslated region (3′UTR) of SOCS3 mRNA (Fig. 6A). The dual luciferase reporter system was then used to confirm miR-222-3p’s regulation on SOCS3 3 in HEK 293T cells. We created luciferase reporter plasmids first. The plasmid having a mutant SOCS3 3′ UTR region was designated pmir-SOCS3-mut while the plasmid holding the wild-type 3′ UTR from SOCS3 was designated pmir-SOCS3 (Fig. 6A). In contrast to cells transfected with the mutant 3′ UTR, transfection of miR-222-3p mimic led to a noticeably lower level of luciferase activity in HEK 293 T cells transfected with pmir-SOCS3, as seen in Fig. 6B. Then, we utilized Western blotting and RT-qPCR to detect SOCS3 protein and mRNA expression in BMM, respectively. The results presented that the SOCS3 protein and mRNA level was significantly decreased in the mimic group (Fig. 6C–E). Furthermore, SOCS3 overexpression rescued the decreased iNOS level and prevented the upregulated Arg-1 level of macrophages caused by miR-222-3p-mimic (Fig. 6F–I). In summary, miR-222-3p inhibits SOCS3 and consequently reverses pro-inflammatory macrophage polarization toward anti-inflammatory macrophage polarization. Finally, we deduce that miR-222-3p stimulates macrophages' transition to an anti-inflammatory state by preventing SOCS3 from functioning.

Through KEGG (https://www.kegg.jp/kegg/) investigation, we discovered that SOCS3 is a negative regulator of the JAK2/STAT3 signaling pathway, which allowed us to further investigate the underlying signaling pathway mechanism of SOCS3. We thus employed Western Blotting to measure the levels of JAK2/STAT3 pathway protein expression in BMMs transfected with miR-222-3p mimic/inhibitor, and we noticed that JAK2/STAT3 pathway was activated in the mimic group while suppressed in the inhibitor group (Fig. 6J–N). Taken together, we confirmed that miR-222-3p functions biologically by targeting the SOCS3/JAK2/STAT3 axis.

### EPC-EXOs-derived miR-222-3p promotes anti-inflammatory macrophages via the SOCS3/JAK2/STAT3 axis in vitro and in vivo and improves the outcome of spinal cord injury

**Fig. 7:**
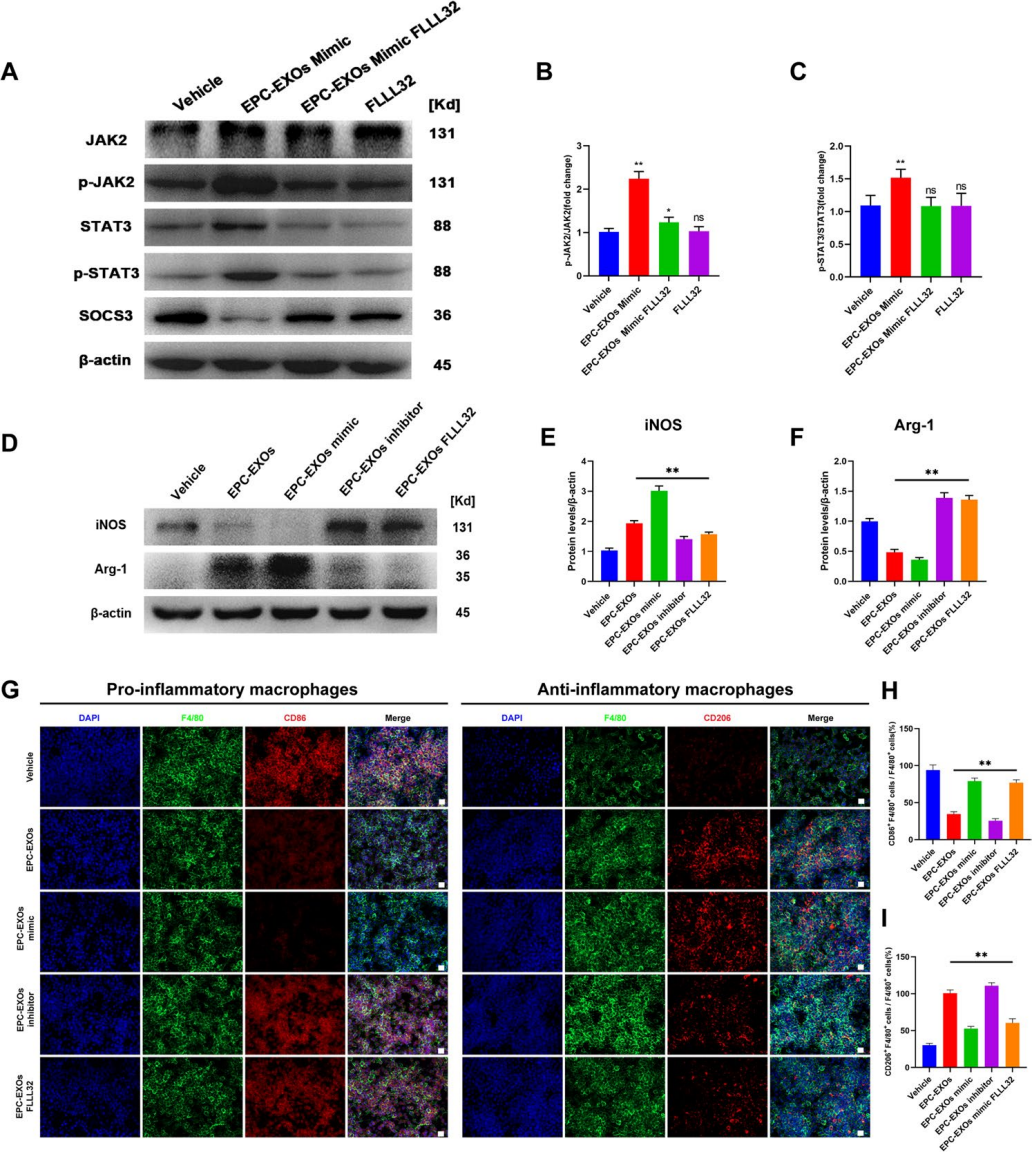

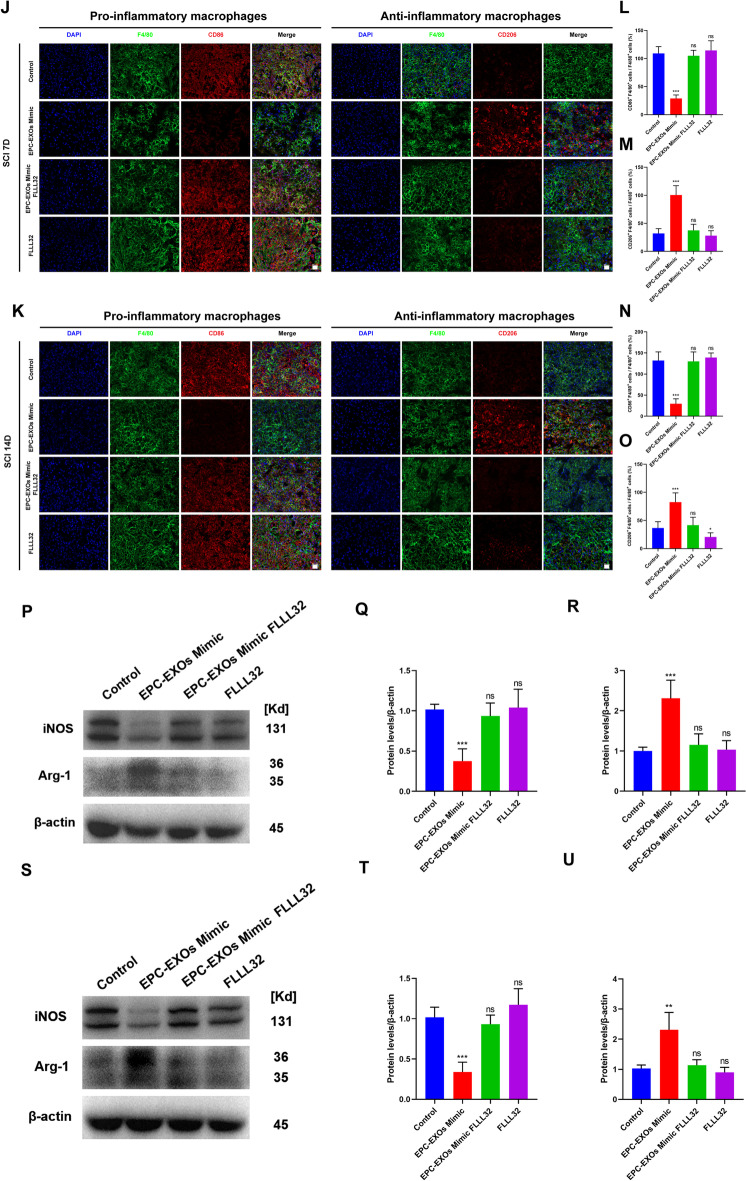

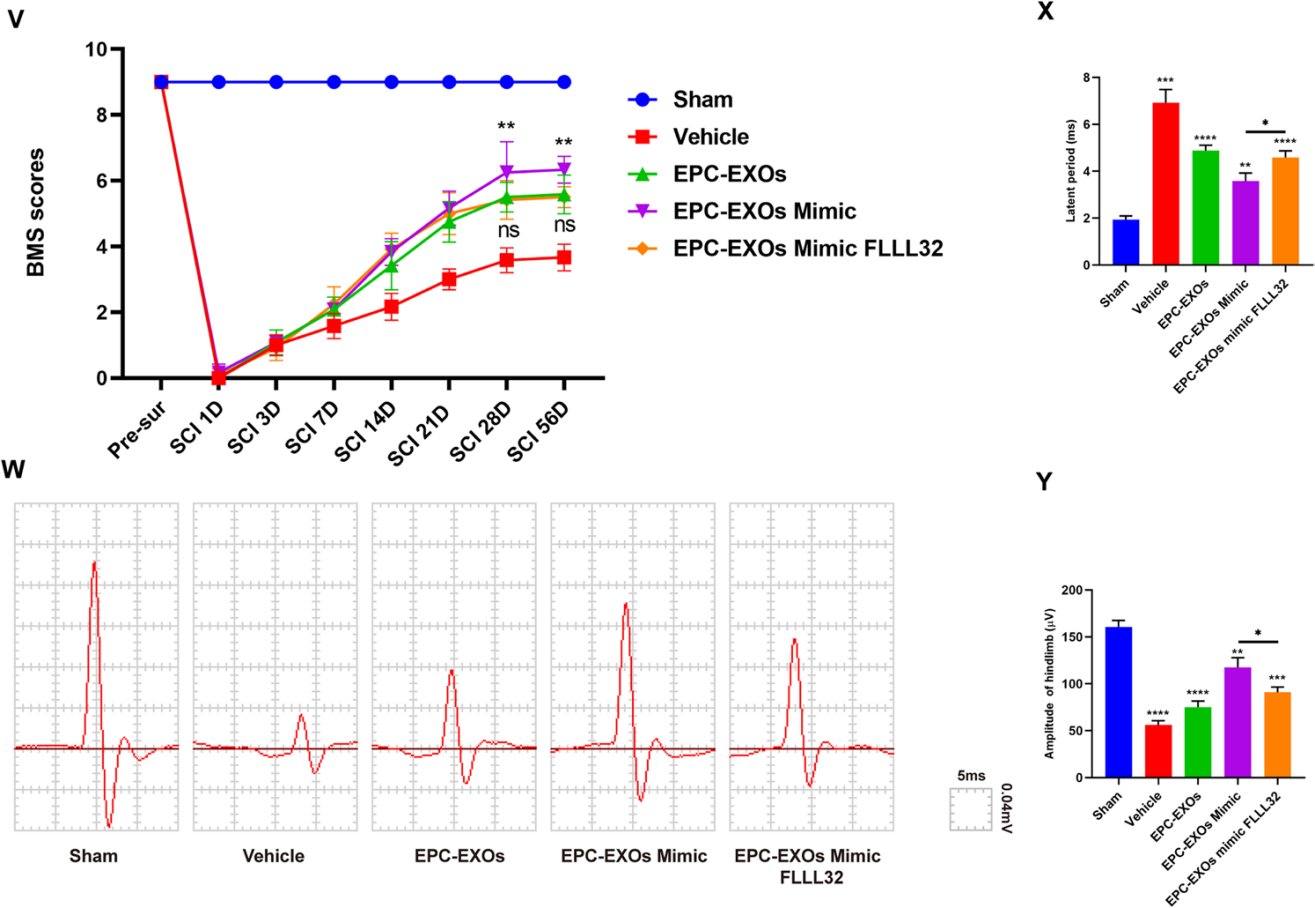
EPC-EXOs-derived miR-222-3p promotes anti-inflammatory macrophages via the SOCS3/JAK2/STAT3 axis in vitro and in vivo and improves the outcome of spinal cord injury. **A** Western Blotting analysis of the expression levels of key proteins of the SOCS3/JAK2/STAT3 signaling pathway (SOCS3, JAK2, p-JAK2, STAT3, and p-STAT3) after EPC-EXOs, FLLL32, and EPC-EXOs + FLLL32 interventions in BMMs. **B**, **C** Statistical analysis of the expression levels of SOCS3, p-JAK2/JAK2 and p-STAT3/STAT3, *n* = 3, ^ns^P > 0.05, *P < 0.05, **P < 0.01. **D** Western Blotting analysis of the expression levels of iNOS and Arg-1 in the BMMs treated with EPC-EXOs, EPC-EXOs Mimic, EPC-EXOs Inhibitor and EPC-EXOs FLLL32. **E**, **F** Statistical analysis of the expression levels of iNOS and Arg-1 in each group, *n* = 3, **P < 0.01. **G** Representative immunofluorescence images of the groups: Vehicle, EPC-EXOs, EPC-EXOs Mimic, EPC-EXOs Inhibitor and EPC-EXOS FLLL32 interfering with CD86/CD206 (red) and DAPI (blue) staining of F4/80^+^ macrophages (green). Scale bar: 50 μm. **H**, **I** Statistical analysis of the proportion of CD86^+^F4/80^+^ cells or CD206^+^F4/80^+^ cells to total F4/80^+^ cells, *n* = 3, **P < 0.01. **J**, **K** Immunofluorescence images of F4/80^+^ macrophages (green), CD86^+^/CD206^+^ (red) and DAPI (blue) staining of spinal cord injury sections of mice representative of the groups: Control, EPC-EXOs Mimic, EPC-EXOs Mimic FLLL32 and FLLL32 at 7- and 14- days post-injury (dpi) in wild-type mice. Scale bar: 20 μm. **L**–**O** Immunofluorescence results were analyzed by ImageJ, GraphPad, and SPSS. *n* = 3, ^ns^*P* > 0.05, *P < 0.05, ***P < 0.001. **P**, **S** Western Blotting detected the expression levels of iNOS and Arg-1 in macrophages in the region of injury at 7 and 14 days post spinal cord injury in the groups: Control, EPC-EXOs Mimic, EPC-EXOs Mimic FLLL32 and FLLL32. **Q**–**U** Statistical analysis of the expression levels of iNOS and Arg-1, *n* = 3, ^ns^*P* > 0.05, **P < 0.01, ***P < 0.001. **V** BMS primary score over time post-SCI in Sham, Vehicle, EPC-EXOs, EPC-EXOs Mimic and EPC-EXOs Mimic FLLL32 group, *n* = 5, ^ns^*P* > 0.05, **P < 0.01. **W** Representative images of motor-evoked potential (MEP) in Sham, Vehicle, EPC-EXOs, EPC-EXOs Mimic and EPC-EXOs Mimic FLLL32 group at 28 days post-SCI. **X**, **Y** Quantification of **W**, *n* = 6, *P < 0.05

To further elucidate whether EPC-EXOs-derived miR-222-3p activates the JAK2/STAT3 signaling pathway and thus plays a role in regulating macrophage polarization, we first examined the expression levels of key proteins of the JAK2/STAT3 signaling pathway in EPC-EXOs transfected with miR-222-3p mimic and FLLL32, a selective small molecule inhibitor of JAK2/STAT3 signaling pathway, interfering with BMMS by Western Blotting. Figure 7A–C shows that adding FLLL32 considerably reversed the trend of EPC-EXOs Mimic that activated the JAK2/STAT3 signaling pathway. Also, the iNOS was downregulated in the EPC-EXOs mimic group and increased in the EPC-EXOs FLLL32 group, while the expression trend of Arg-1 was opposite (Fig. 7D–F). Moreover, these results were confirmed by the subsequent immunofluorescence labeling assays (Fig. 7G–I), which showed that FLLL32 dramatically reduced the propensity of EPC-EXOs to reverse the polarization of macrophages toward pro-inflammation. Overall, EPC-EXOs-derived miR-222-3p boosted macrophage polarization toward anti-inflammation and JAK2/STAT3 blockage depressed this effect.

Further, we attempted to investigate whether EPC-EXOs-derived miR-222-3p regulated macrophage polarization dependent on JAK2/STAT3 pathway activation. As the immunofluorescence results presented (Fig. 7J–O), EPC-EXOs transfected with miR-222-3p mimic dramatically enhanced the proportion of CD206^+^F4/80^+^ macrophages while decreasing the fraction of CD86^+^F4/80^+^ macrophages, and these effects were blocked entirely by additional FLLL32 treatment. This was further confirmed by Western Blotting (Fig. 7P–U), which showed that on both day 7 and day 14 after injury, selective small molecule inhibitors of the JAK2/STAT3 signaling pathway redeemed the original role of miR-222-3p mimic in promoting elevated expression of Arg-1 levels while decreasing expression of iNOS levels, indicating that the role of EPC-EXOs-derived miR-222-3p in macrophage polarization relied on JAK2/STAT3 pathway activation.

Since the miR-222-3p/JAK2/STAT3 axis was validated in vitro, we then explored whether this axis activation could improve animal motor behavior. We compared the BMS scores of the EPC-EXOs, EPC-EXOs Mimic, and EPC-EXOs Mimic in conjunction with FLLL32 groups to those of the Vehicle group, and discovered that these groups had considerably higher BMS scores (Fig. 7V). The tail vein injection of EPC-EXOs Mimic achieved a better score, lower latent period, and higher amplitude, and the addition of FLLL32 attenuated this trend (see Fig. 7W–Y).

**Fig. 8:**
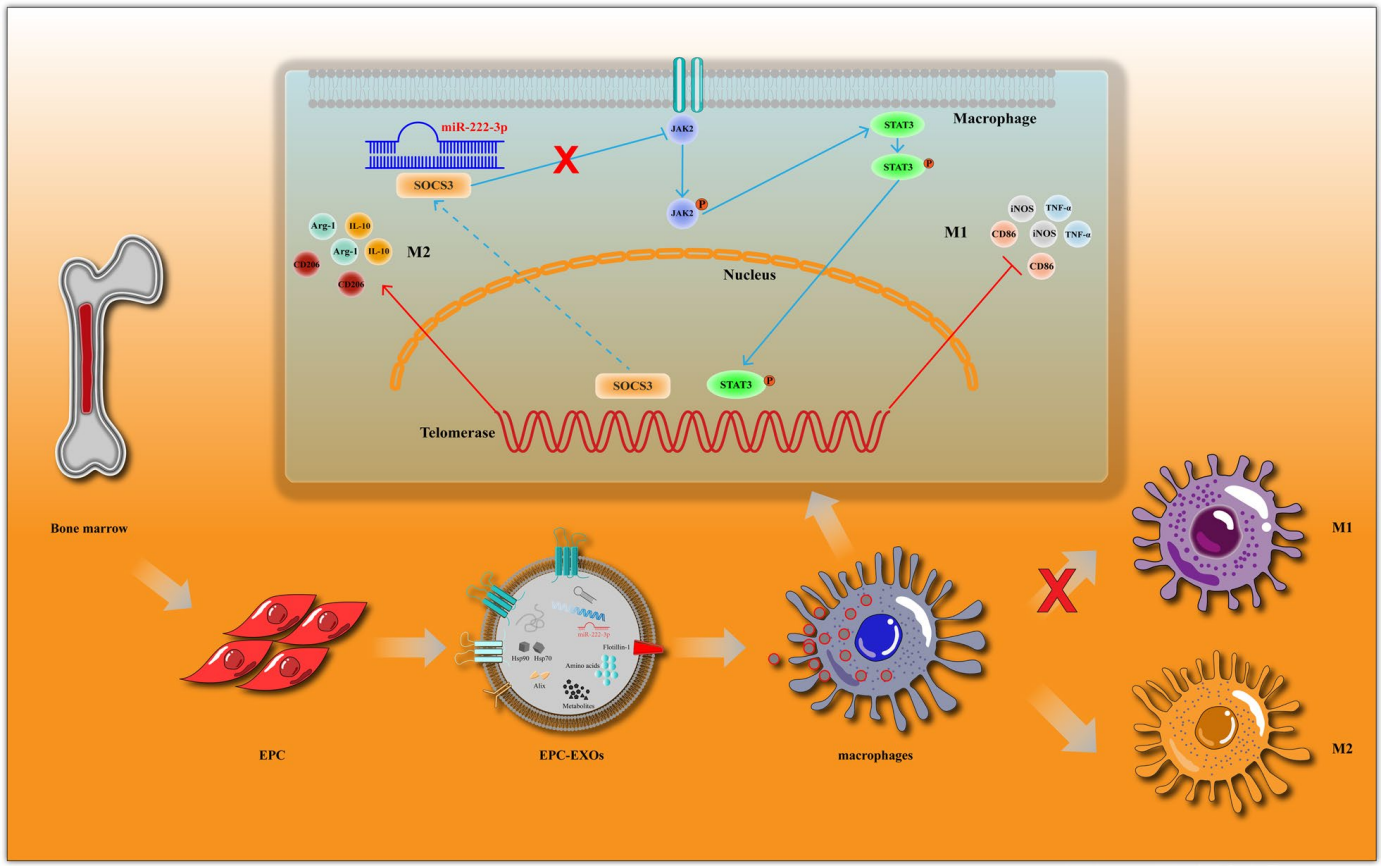
MiR-222-3p of bone marrow-derived EPC-EXOs regulates the polarization of macrophages via the SOCS3/JAK2/STAT3 axis after SCI

All of the aforementioned findings point to the conclusion that miR-222-3p, which is produced from EPC-EXOs, promotes macrophage polarization toward anti-inflammatory macrophages by activating the SOCS3/JAK2/STAT3 signaling pathway. Eventually, the neurological function after spinal cord injury is well improved (Fig. 8).

## Discussion

Spinal cord injury (SCI) is still a difficult problem worldwide [52, 53]. The main challenge is the inability of nerve axons to regenerate, leading to a decline in nerve function or even loss of function [54]. The current approach to treating spinal cord injuries still focuses on swelling control, pain management, hyperbaric oxygen therapy, and nerve nourishment but ignores the underlying problem of axonal regrowth [55–57]. Insufficient numbers of endogenous stem progenitor cells and regenerative potential [54, 58], a lack of blood flow [43], a lack of auxiliary scaffolding to support nerve axon regeneration [59], inhibition of complex microenvironmental components [60], and the obstructive effect of scarring [61–63] are all factors that have been identified as contributing to the failure to regenerate nerve axons after spinal cord injury. Based on these study foundations, an increasing number of scientists are investigating therapeutic strategies at the micro level, including the use of small molecule medicines [64, 65], the manufacturing of 3D biomaterials [66, 67], and the transfer of exogenous stem cells [58], all of which have shown promising results.

In this study, we purified the exosomes secreted by bone marrow-derived endothelial progenitor cells for transplantation in spinal cord injury. The experimental findings demonstrated that EPC-EXOs do indeed play a role in regulating the phenotypic changes of immune cells in the injured area and fostering the regeneration of nerve axons. Endothelial progenitor cells are a newly discovered stem progenitor cell type that have received little attention in the field of spinal cord injury. However, they have received more attention in the fields of cardiac tissue regeneration and repair [68, 69], sepsis treatment [26], and respiratory system [70]. These studies have been conducted primarily to support physiological effects like vascular regeneration and inflammation control, which are essential in spinal cord injury. It would therefore be definite interesting to learn more about these biological effects. However, a large number of laboratory and clinical studies have discovered that the sole transplantation of stem progenitor cells entails an inherent risk of tumorigenic and immune rejection, which can be extremely dangerous to the patient [17, 18, 50]. Exosomes are cellular or eukaryotic-derived microvesicles that can potentially be more effective than traditional drug interventions and cell transplantation because they carry biologically active molecules like DNA, RNA, proteins, and lipids present in the host cell. As a result, to avoid the potential risk, we combined this hotspot of research. Compared to general drug treatments and cell transplantation, they also have the benefits of readily passing the blood–spinal cord barrier and being highly targeted, significantly expanding their use. Exosome subpopulations need to be finely divided, according to some scientists, in order to fully and thoroughly understand the biological nature of the various subpopulations of exosomes because the differences between exosomes secreted by different cells or tissues and the identification of exosomes secreted by the same cell or tissue at different stages cannot be explained by current research on exosomes. Furthermore, the mechanism of exosome recognition and targeted uptake by various target receptor cells has not been fully elucidated. Exosome transplantation is plagued by numerous dose, timing, and activity maintenance issues, which seems to offer more ideas for future study.

The long-term infiltration of mononuclear macrophages and microglia activation at the site of spinal cord injury is one of the key factors contributing to progressive tissue degeneration and difficulties in axonal regeneration, and how to effectively control the immune cells and immune microenvironment in the area of injury has been the subject of many studies [71–74]. Previous studies have also investigated how various measures to suppress the inflammatory environment and immune cell phenotypic changes can facilitate recovery from spinal cord injury. Additionally, exosomes from M2 microglia or macrophages have been used in our earlier studies and those of other researchers to successfully polarize immune cells in situ and reconstruct neurovascular units [43]. Exosomes have also been used to transport drugs or for basic inhibition in these studies [64]. There is controversy surrounding the phenotypic transformation of immune cells (such as macrophages and microglia), ranging from the traditional dichotomy (M1-pro-inflammatory and M2-anti-inflammatory) to more recent studies that use a “Spectrum Model” to categorize the M1-type based on IFN-γ intervention and the M2-type based on IL-4 intervention [75]. The key argument is that these immune cells' phenotypes are dynamic rather than static, with the conventional M1 and M2 subtypes serving as the two extremes of a fully polarized immune cell and that these two phenotypes are frequently symbiotic and reciprocal. The primary question in this experiment, which was not only entirely polarized, was whether the macrophage phenotype favored a pro-inflammatory or an anti-inflammatory state after the administration of EPC-EXOs. Naturally, with additional research, we will be able to more accurately categorize the phenotypic change of immune cells in conjunction with the “Spectrum Model” and delve deeper into the process. Furthermore, whether polarized macrophages were already polarized before the injury and were subsequently recruited to the injured area or whether mononuclear macrophages infiltrated the injured area and underwent phenotypic change as a result of the injury microenvironment is still up for debate. This needs further explanation and will likely require future research [76].

EPCs express both stem cell markers (CD133 or CD34) and endothelial cell markers because they have the properties of stem cells that self-renew and the capacity to develop into mature endothelial cells (VEGFR2 or CD31). Notably, the expression levels of CD31 and CD133 in EPC's cellular markers varied at different stages. In this study, the majority of the EPCs used in our experiment were early-stage cells, allowing us to fully make use of their stem cell features [77, 78].

Prior research has focused on miR-222-3p with a bias for internal tumor processes that support cell growth, block apoptosis, and support TAM transition to the M2-like phenotype [37, 79]. However, very little research on spinal cord injury has been published. This study discovered that miR-222-3p promoted anti-inflammatory polarization and contributed to the SCI repair for the first time.

It is well known that the mechanism of miRNA regulation of target genes is mainly through binding to the 3′UTR of the downstream target gene mRNA, thus silencing the mRNA and further hindering the translation process of the target gene [80]. Since SOCS3 is one of the critical factors regulating macrophage polarization [37, 38, 40] manipulated the expression of miR-222-3p and found that miR-222-3p mimic could significantly inhibit the expression of SOCS3, indicating SOCS3 as a downstream target gene of miR-222-3p. This was further confirmed by miR-222-3p and SOCS3 binding prediction and dual luciferase reporter assay. Besides, previous research has shown that SOCS3 functions as a negative feedback regulator of the JAK2/STAT3 signaling pathway [37], and this naturally arose a hypothesis that miR-222-3p might activate JAK2/STAT3 pathway by inhibiting SOCS3, and it also experimentally verified in our study. Hence, we finally confirmed that miR-222-3p regulates the SOCS3/JAK2/STAT3 axis to facilitate macrophage polarization regulation. This discovery is novel for it revealed the underlying mechanism of miR-222-3p-mediated macrophage polarization, and will provide a novel target for converting macrophage phenotype to promote SCI repair.

Since most studies of signaling pathways have used small molecule inhibition in response to experimental validation, small molecule inhibitors [81–83] were also chosen for this experiment to investigate the signaling pathway. However, whether the small molecule inhibitors act on pre-selected macrophages when administered in vivo raises concerns about the study's design. Did additional organs and cells respond to the injection of the small molecule inhibitor? We will attempt to optimize the experimental technique in future investigations to improve the data’s accuracy, but these questions cannot be ignored.

## Conclusion

We discovered that EPC-EXOs derived miR-222-3p transferred macrophages at the SCI area from pro-inflammatory (M1-like) phenotype to anti-inflammatory (M2-like) phenotype in vitro and improve the motor behavior of mice via tail vein injection. Mechanically, miR-222-3p was confirmed to suppress SOCS3 and activate the downstream JAK2/STAT3 pathway, and its effects on macrophage polarization were verified as JAK2/STAT3 pathway-dependent. This reveals the mechanism for macrophage polarization by intercellular exosome communication and will also provide a novel strategy to promote damage repair after SCI.

## Supplementary Information

**Additional file 1: Figure S1.** The morphology of BMMs and EPCs under optical microscope. Representative the morphology of the mature macrophages. Scale bar: 20 μm.Representative colony morphology of early EPCs. Scale bar: 200 μm. **Figure S2.** Flow cytometry analysis of the EPCs’ specificity markersare highly expressed. **Figure S3.** Statistics on the uptake of Dil-labeled exosomesby macrophagesin the injured region after spinal cord injury, *n* = 3. **Figure S4.** EPC-EXOs promote axonal regeneration after spinal cord injury and inhibit apoptosis in the injured region.Immunofluorescence images of Tuj1 axonand DAPIstaining of spinal cord injury sections of mice representative of the Sham group, Control group and EPC-EXOs group at 28 dpi in wild-type mice. The white dashed area is the core of the spinal cord injury. Scale bar: 200 µmImmunofluorescence images of apoptosis staining-TUNELand DAPIstaining of spinal cord injury sections of mice representative of the Sham group, Control group and EPC-EXOs group at 3 dpi in wild-type mice. Scale bar: 100 µmImmunofluorescence results were analyzed by ImageJ, GraphPad, and SPSS, *n* = 3, **P < 0.01. **Figure S5.** qRT-PCR analysis of pri-miRNA expression in macrophages treated with EPC-EXOs or not, *n* = 3, ^ns^P > 0.05. **Table S1.** qRT-PCR primer sequences in this study.

## Data Availability

All raw data in this manuscript are available on request. The original contributions presented in the study are included in the article/additional file, further inquiries can be directed to the corresponding author/s.
