# Supplementary material for: Endothelial progenitor cell-derived exosomes promote anti-inflammatory macrophages via SOCS3/JAK2/STAT3 axis and improve the outcome of spinal cord injury
Source: J Neuroinflammation. 2023 Jun 30;20:156. doi: 10.1186/s12974-023-02833-7 (PMC10314438; doi:10.1186/s12974-023-02833-7)
Supplement: Supplementary file 1 — Additional file 1: Figure S1. The morphology of BMMs and EPCs under optical microscope. Representative the morphology of the mature macrophages. Scale bar: 20 μm.Representative colony morphology of early EPCs. Scale bar: 200 μm. Figure S2. Flow cytometry analysis of the EPCs’ specificity markersare highly expressed. Figure S3. Statistics on the uptake of Dil-labeled exosomesby macrophagesin the injured region after spinal cord injury, n = 3. Figure S4. EPC-EXOs promote axonal regeneration after spinal cord injury and inhibit apoptosis in the injured region.Immunofluorescence images of Tuj1 axonand DAPIstaining of spinal cord injury sections of mice representative of the Sham group, Control group and EPC-EXOs group at 28 dpi in wild-type mice. The white dashed area is the core of the spinal cord injury. Scale bar: 200 µmImmunofluorescence images of apoptosis staining-TUNELand DAPIstaining of spinal cord injury sections of mice representative of the Sham group, Control group and EPC-EXOs group at 3 dpi in wild-type mice. Scale bar: 100 µmImmunofluorescence results were analyzed by ImageJ, GraphPad, and SPSS, n = 3, **P < 0.01. Figure S5. qRT-PCR analysis of pri-miRNA expression in macrophages treated with EPC-EXOs or not, n = 3, nsP > 0.05. Table S1. qRT-PCR primer sequences in this study. [file 12974_2023_2833_MOESM1_ESM.docx]

**Additional file 1: Materials**

**
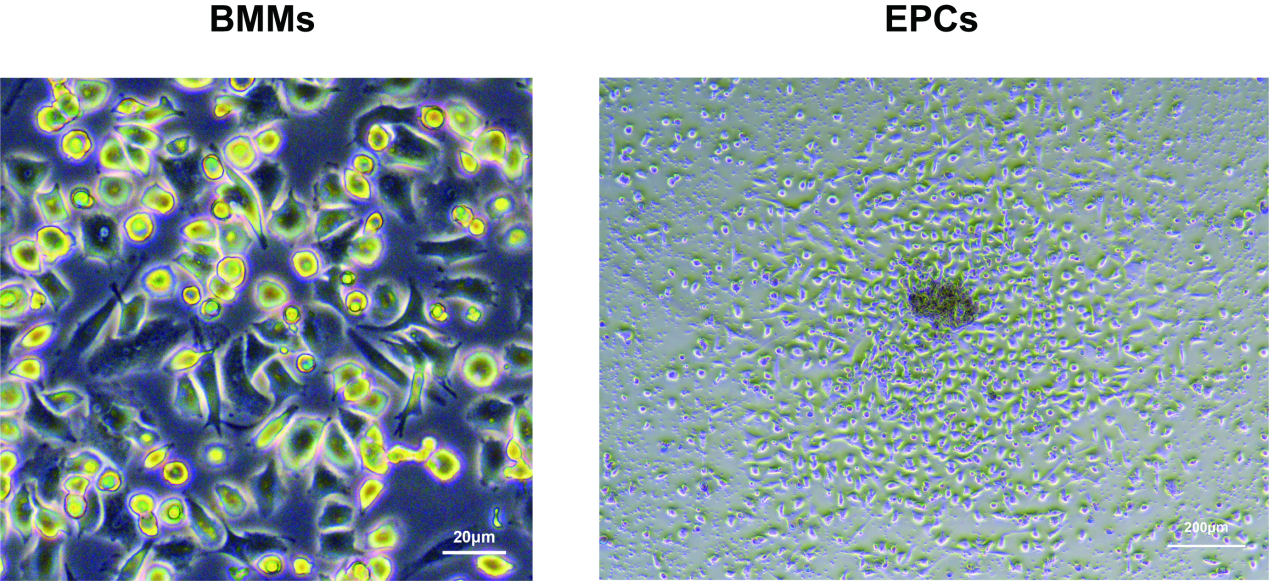
**

**Figure S1. The morphology of BMMs and EPCs under optical microscope. (A)** Representative the morphology of the mature macrophages. Scale bar: 20μm. (B)

Representative colony morphology of early EPCs. Scale bar: 200μm.


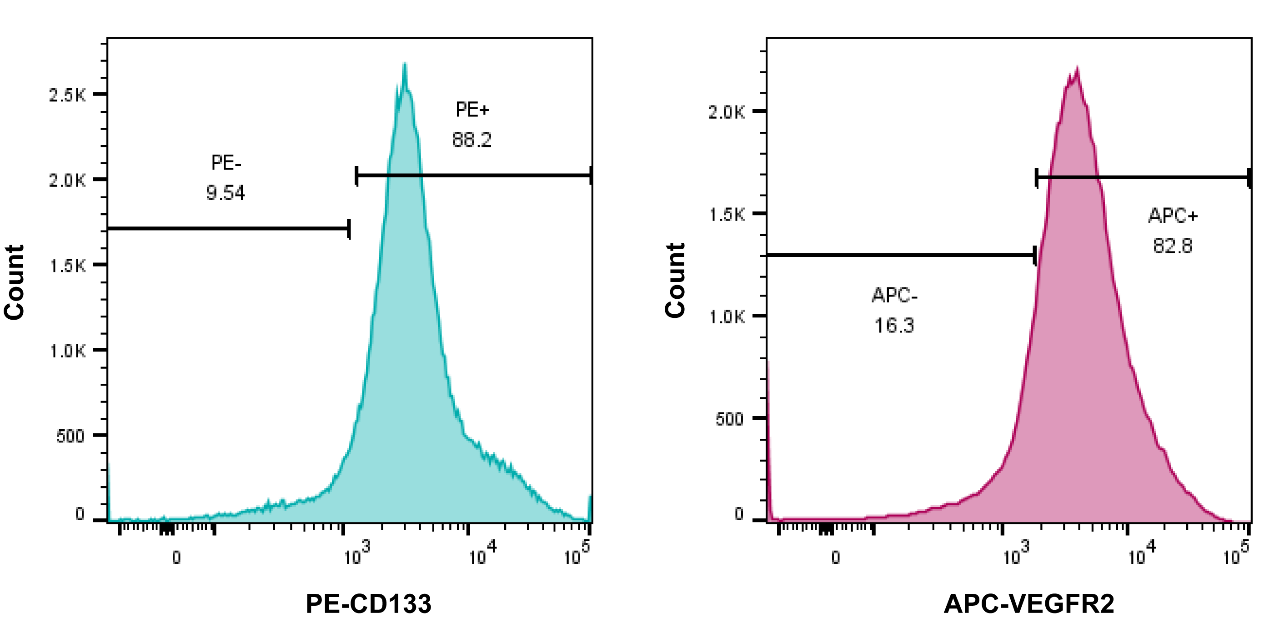


**Figure S2. Flow cytometry analysis of the EPCs’ specificity markers (CD133 and VEGFR2) are highly expressed.**


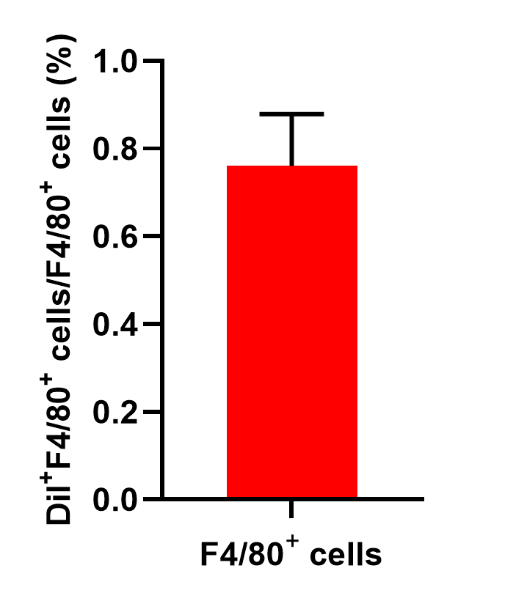


**Figure S3. Statistics on the uptake of Dil-labeled exosomes (red) by macrophages (F4/80^+^) (green) in the injured region after spinal cord injury, n=3.**


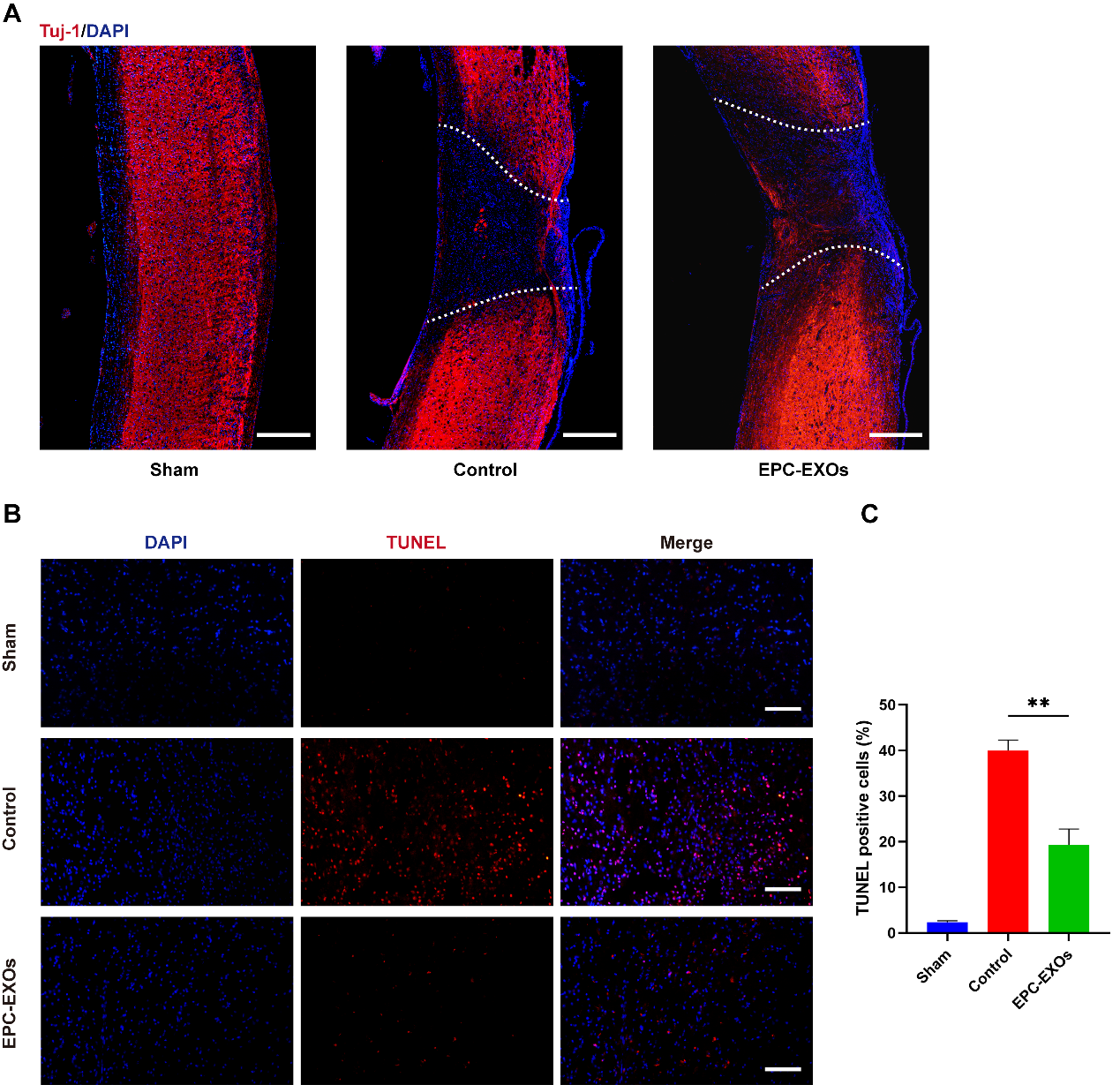


**Figure S4. EPC-EXOs promote axonal regeneration after spinal cord injury and inhibit apoptosis in the injured region.** (A) Immunofluorescence images of Tuj1 axon (red) and DAPI (blue) staining of spinal cord injury sections of mice representative of the Sham group, Control group and EPC-EXOs group at 28 dpi in wild-type mice. The white dashed area is the core of the spinal cord injury. Scale bar: 200µm (B) Immunofluorescence images of apoptosis staining-TUNEL (red) and DAPI (blue) staining of spinal cord injury sections of mice representative of the Sham group, Control group and EPC-EXOs group at 3 dpi in wild-type mice. Scale bar: 100µm (C) Immunofluorescence results were analyzed by ImageJ, GraphPad, and SPSS, n=3, **P<0.01


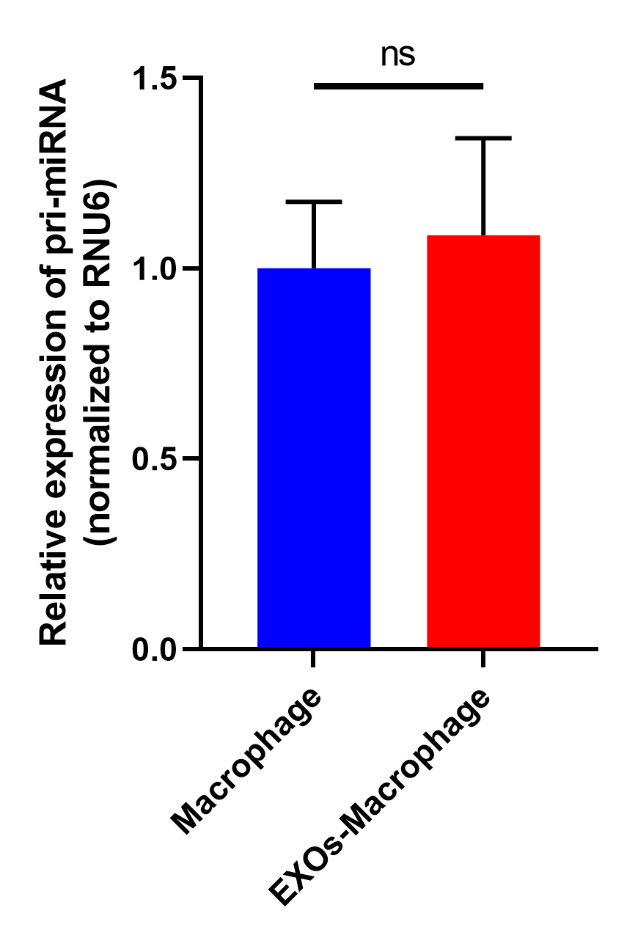


**Figure S5. qRT-PCR analysis of pri-miRNA expression in macrophages treated with EPC-EXOs or not, n=3, ^ns^P>0.05.**

**Table S1. qRT-PCR primer sequences in this study**

| Gene | Primer sequence 5’- 3’ | |
| --- | --- | --- |
|  | Forward | Reverse |
| iNOS | GTTCTCAGCCCAACAATACAAGA | GTGGACGGGTCGATGTCAC |
| TNF-α | CAGGCGGTGCCTATGTCTC | CGATCACCCCGAAGTTCAGTAG |
| CD86 | TCAATGGGACTGCATATCTGCC | GCCAAAATACTACCAGCTCACT |
| Arginase-1 | CTCCAAGCCAAAGTCCTTAGAG | GGAGCTGTCATTAGGGACATCA |
| IL-10 | CTTACTGACTGGCATGAGGATCA | GCAGCTCTAGGAGCATGTGG |
| CD206 | CTCTGTTCAGCTATTGGACGC | TGGCACTCCCAAACATAATTTGA |
| SOCS3 | ATGGTCACCCACAGCAAGTTT | TCCAGTAGAATCCGCTCTCCT |
| GAPDH | AGGTCGGTGTGAACGGATTTG | GGGGTCGTTGATGGCAACA |
